# Efficacy of methimazole combined with traditional Chinese medicine in the treatment of Graves’ disease: a systematic review and network meta-analysis

**DOI:** 10.3389/fendo.2026.1767935

**Published:** 2026-07-14

**Authors:** Tiantian Kuan, Wenzhi Tian, Peiran Wang, Dong Chen, Chenchen Hu, Peng Li, Xi Su

**Affiliations:** 1 Department of Thyroid and Breast Surgery, Peking University Shenzhen Hospital, Peking University (PKU)-Shenzhen Clinical Institute of Shantou University Medical College, Shenzhen, China; 2 Shenzhen University Clinical Medical Academy Center, Shenzhen University, Shenzhen, Guangdong, China; 3 Department of Thyroid and Breast Surgery, Peking University Shenzhen Hospital, Shenzhen, Guangdong, China

**Keywords:** Graves’ disease, methimazole, network meta-analysis, systematic review, traditional Chinese medicine

## Abstract

**Introduction:**

Traditional Chinese Medicine (TCM) Combined with Methimazole (MMI) versus MMI Alone for Thyroid Function and Autoantibodies in Graves’ Disease(GD).

**Methods:**

A systematic literature search was performed in PubMed, Cochrane Library, Web of Science, EMBASE, CNKI, VIP, CBM, and Wanfang databases up to June 2025 for RCTs comparing TCM plus MMI versus MMI alone. Study risk of bias was evaluated using the Cochrane tool. We assessed transitivity to ensure intervention comparability, verified consistency via node-splitting analysis, and planned sensitivity analysis according to risk-of-bias classification. A network meta-analysis was conducted in Stata 15.1 using a random-effects model. Results are reported as mean differences (MD) or standardized mean differences (SMD) with 95% confidence intervals (CI), and treatment efficacy was ranked using SUCRA values.

**Results:**

A network meta-analysis of 15 RCTs involving 1,317 GD patients and 14 TCM plus MMI regimens showed potential advantages over MMI alone in improving thyroid function indices and reducing selected autoantibodies, particularly TRAb and TPOAb. Adverse events were summarized descriptively because AE reporting was incomplete and heterogeneous across the included RCTs. Regarding SUCRA rankings, the Modified Huagan Decoction (MHD) + MMI ranked first for reducing FT3 (SUCRA = 93.4%), the Modified Xiaoyao Powder (MXYP) + MMI ranked first for reducing FT4 (SUCRA = 97.3%), and the Xiaoyao Powder (XYP) + MMI ranked first for regulating TSH (SUCRA = 96.3%). For decreasing TRAb, the MHD + MMI regimen ranked first (SUCRA = 88.2%), while for lowering TPOAb, the Huotan Jiangni Formula (HJF) + MMI showed the highest SUCRA ranking (SUCRA = 100%).

**Conclusion:**

TCM combined with MMI may have beneficial effects on thyroid function and selected autoantibody levels, particularly TRAb and TPOAb, in GD patients versus MMI alone. Among the evaluated regimens, MHD + MMI, MXYP + MMI, XYP + MMI, and HJF + MMI showed high SUCRA rankings for specific outcomes, including FT3, FT4, TSH, TRAb, and TPOAb. These findings suggest that TCM plus MMI may be a promising short-term adjunctive strategy for initial GD treatment, although safety evidence remains limited by incomplete and inconsistent AE reporting.

**Systematic review registration:**

https://www.crd.york.ac.uk/prospero/, identifier CRD420251151307.

## 1 Introduction

GD is the leading cause of hyperthyroidism. In iodine-sufficient regions, GD accounts for 70%–80% of hyperthyroidism cases, whereas in iodine-deficient areas, the proportion declines to about 50%. GD occurs worldwide, most commonly between the ages of 30 and 50, and shows a clear female predominance. Currently, the main Western medical treatments for GD include antithyroid drug therapy, radioactive iodine-131 (¹³¹I) therapy, and thyroidectomy. (1, 2) Antithyroid drugs are associated with a variety of adverse reactions. Common adverse effects include agranulocytosis, fever, skin rashes, urticaria, arthralgia, and drug-induced liver injury. (3) TCM is increasingly recognized as a promising therapy for GD, as it can enhance efficacy, shorten treatment duration, and address the limitations of Western medicine. (4) Importantly, the potential therapeutic advantages of combining TCM with MMI remain largely unexplored. Most existing studies have focused on MMI or TCM monotherapy, with limited attention given to their potential synergistic effects. Different combinations of TCM and MMI exhibit varying therapeutic efficacy. This meta-analysis evaluates the short-term efficacy of TCM plus MMI in newly diagnosed overtly hyperthyroid GD patients, not long-term remission or relapse in euthyroid patients. A network meta-analysis was conducted by synthesizing data from RCTs comparing TCM combined with MMI versus MMI alone, focusing on their efficacy in reducing thyroid autoantibodies (TRAb, TPOAb, TgAb) and improving thyroid function indicators (FT3, FT4, and TSH). Meta-analysis is an evidence-based approach that enables direct and indirect comparisons of multiple interventions to evaluate and rank their therapeutic efficacy. (5) Consequently, this study compared the therapeutic effects of multiple TCM formulations—including Xiehuo Xiaoying Formula (XHF), Xiaoyao Powder (XYP), Modified Huagan Decoction (MHD), Yueju Pill (YJP), Shugan Xiajia Formula (SXF), Sanjie Xiaoying Decoction (SXD), Ziyin Xiaoying Formula (ZXF), Longmu Xiaoyao Powder (LMXP), Huotan Jiangni Formula (HJF), Modified Xiaoyao Powder (MXYP), Astragalus membranaceus Preparations (AMP), Chaihu Shaoyao Decoction (CSD), Yingliu Mixture (YLM), and Haizao Yuhu Decoction (HYD)—for the treatment of patients with GD. This study aimed to assess the therapeutic effects of combining Chinese medicinal preparations with MMI, to provide more effective treatment options.

## 2 Methods

This systematic review and network meta-analysis was conducted in accordance with the Preferred Reporting Items for Systematic Reviews and Meta-Analyses extension statement for network meta-analyses (PRISMA-NMA) and methodological guidance from the Cochrane Handbook.

### 2.1 Search strategy

We searched eight electronic databases, including PubMed, Cochrane Library, Web of Science, EMBASE, China National Knowledge Infrastructure (CNKI), VIP, Chinese Biomedical Database (CBM), and Wanfang, from database inception to June 2025. The search strategy was constructed around the PICOS tool: (P) Population: patients with Graves’ disease; (I) Intervention: TCM combined with MMI; (C) Comparison: MMI (O) Outcome metrics: FT3, FT4, TSH, TRAb, TPOAb, TgAb; and (S) Type of study: RCT. The detailed search strategy is shown in **Table 1** (using PubMed as an example).

**Table 1:** Search strategies on PubMed.

No.	Search strategy
#1	“Graves Disease”[Mesh]
#2	(((((((((((((Disease, Graves)) OR (Basedow Disease)) OR (Disease, Basedow)) OR (Hyperthyroidism, Autoimmune)) OR (Hyperthyroidism, Autoimmune)) OR (Exophthalmic Goiters)) OR (Goiters, Exophthalmic)) OR (Goiter, Exophthalmic)) OR (Graves’ Disease)) OR (Disease, Graves’)) OR (Basedow’s Disease)) OR (Basedows Disease)) OR (•Disease, Basedow’s)
#3	#1 OR #2
#4	“Medicine, Chinese Traditional”[Mesh]
#5	(((((((((((((Zhong Yi Xue) OR (Chung I Hsueh)) OR (Hsueh, Chung I)) OR (Traditional Medicine, Chinese)) OR (Chinese Traditional Medicine)) OR (TCM)) OR (Chinese Medicine, Traditional)) OR (Traditional Tongue Diagnosis)) OR (Tongue Diagnoses, Traditional)) OR (Tongue Diagnosis, Traditional)) OR (Traditional Tongue Diagnoses)) OR (Traditional Tongue Assessment)) OR (Tongue Assessment, Traditional)) OR (Traditional Tongue Assessments)
#6	#4 OR #5
#7	“Methimazole”[Mesh]
#8	(((((((((((((((((((((((Methymazol) OR (1-Methyl-2-mercaptoimidazole)) OR (1 Methyl 2 mercaptoimidazole)) OR (Merkazolil)) OR (Methylmercaptoimidazole)) OR (Thiamazole)) OR (Thimazol)) OR (Mercasolyl)) OR (Tiamazol)) OR (Mercazolyl)) OR (Tapazole)) OR (Metisol)) OR (Metizol)) OR (Mercazol)) OR (Mercazole)) OR (Favistan)) OR (Methizol)) OR (Strumazol)) OR (Thiamazol Henning)) OR (Henning, Thiamazol)) OR (Thiamazol Hexal)) OR (Hexal, Thiamazol)) OR (Thyrozol)) OR (Tirodril)
#9	#7 OR #8
#10	#3 AND #6 AND #9

### 2.2 Inclusion criteria

1. The type of trial design must be a clinical randomized controlled trial, not limited by the use of allocation concealment and blinding.

2. Trial participants were patients with a confirmed diagnosis of active Graves’ disease (GD).

3. Western medical diagnostic criteria referred to the Chinese Guidelines for the Diagnosis and Treatment of Thyroid Diseases prepared by the Endocrine Society of the Chinese Medical Association in 2008. The diagnostic criteria for Graves’ disease were as follows: (1) clinical symptoms and signs of hyperthyroidism; (2) diffuse enlargement of the thyroid gland, confirmed by palpation or ultrasound, which may be absent in a minority of cases; (3) decreased serum TSH concentration and elevated thyroid hormone levels; (4) proptosis or other infiltrative eye signs; (5) pretibial myxedema; and (6) TRAb or TSAb positivity. Among these criteria, items (1)–(3) were necessary for diagnosis, whereas items (4)–(6) were auxiliary diagnostic criteria.

4. Interventions

Control group: MMI + basic treatment (low iodine, high vitamin, high calorie diet).
Experimental group: internal type of Chinese medicine preparation was added to the control group, with consistent MMI dosage and basic treatment with the control group.

5. Outcome indicators should include at least one of the following: Reported as mean ± SD or with sufficient data for effect-size calculation: FT3, FT4, TSH, TgAb, TPOAb, TRAb.

6. The trial presented no statistically significant differences in comparisons of explicit baselines (sex, age, disease duration, baseline thyroid function and autoantibody levels).

### 2.3 Exclusion criteria

(1) The types of trial designs were animal experiments, non-randomized controlled trials, review articles, and clinical experience summaries, as well as conference abstracts with insufficient detailed data. (2) The subject of the study was complications arising from GD. (3) The trial’s intervention did not meet the treatment grouping requirements: the trial group was a randomized controlled trial of herbal medicine plus propylthiouracil (PTU), or herbal medicine combined with acupuncture or other Chinese medical treatments versus Western medicine, or a before-and-after control of itself with herbal medicine alone. (4) The outcome treatment index of the trial did not include one of FT3, FT4, TSH, TgAb, TPOAb or TRAb, or the data was insufficient for effect-size calculation. (5) Comparisons to baseline were not made in the trial, or there were statistically significant differences in baseline characteristics between groups.

### 2.4 Study selection

Literature was screened and excluded using the literature management software Zotero. Two researchers (Tiantian Kuan and Wenzhi Tian) each performed an initial screening of titles to exclude duplicates, non-randomized controlled trials, reviews, conferences, protocols, and correspondence. Subsequently, the abstracts of the remaining articles were read by each of the two researchers (Kuan Tiantian and Tian Wenzhi) to determine which articles should be included and which should be excluded, and then the full text of the selected articles were read by each of the above two researchers and further assessed for inclusion. Throughout the process, the researchers screened the literature independently and discussed and resolved any disagreements in the process through a third researcher (Peiran Wang). A PRISMA flow diagram was generated to fully report the literature screening process and exclusion reasons.

### 2.5 Data extraction

A standardized pre-selected seven-item data extraction form was used to record data from included studies under the following headings: (1) authors, (2) year of publication, (3) country, (4) period of the study, (5) sample size, (6) mean age (± SD) of the study population, and (7) detailed information of TCM and MMI interventions (formulation, dosage, administration frequency, treatment duration), as well as adverse event type and incidence. Two independent researchers conducted data extraction, and discrepancies were resolved by a third researcher. For adverse-event data, we extracted AE reporting status, AE type, number of events in the TCM+MMI and MMI-only groups, severe adverse events, and withdrawals due to AEs. Studies without explicit AE information were coded as “not reported” rather than “no adverse events”; studies explicitly reporting no AEs or normal safety indicators were coded as “explicitly no AE.”

### 2.6 Risk of bias in individual studies

Two investigators independently assessed risk of bias (ROB), assessing the ROB tool in randomized controlled trials according to the Cochrane Handbook version 5.1.0. The following seven areas were considered: (1) randomized sequence generation, (2) treatment allocation concealment, (3) blinding of participants and (4) personnel, (5) completeness of outcome data, (6) selective reporting and (7) other sources of bias. Each domain was rated as low, high, or unclear risk of bias. Trials were categorized into three levels of ROB based on the number of components with potentially high ROB: high risk (5 or more), moderate risk (3 or 4), and low risk (2 or fewer). (6) A risk-of-bias summary plot was generated to visualize the assessment results.

### 2.7 Transitivity assessment for network meta-analysis

Transitivity assumptions, a prerequisite for valid network meta-analysis, were formally evaluated to ensure consistency across direct and indirect comparisons. We assessed the homogeneity of three key dimensions across all included studies: (1) Patient characteristics: consistent GD diagnostic criteria, no significant differences in age, sex, disease duration, and baseline thyroid function/autoantibody levels; (2) Intervention protocols: MMI as the core Western medicine intervention (with basic treatment consistency where applicable), TCM administered as oral preparations (decoctions/powders/granules); (3) Outcome assessments: consistent detection methods and reference ranges for thyroid function/autoantibody indices, with outcome assessments conducted within broadly comparable short-term treatment periods. Transitivity was confirmed if no critical heterogeneity was identified across these dimensions.

### 2.8 Data analysis

In the study where “TCM combined with MMI” was the intervention, all outcome indicators were continuous variables, expressed as “mean ± standard deviation (SD)”. (7) Continuous variables were reported as mean differences (MDs) or standardized mean differences (SMDs) with 95% confidence intervals (CIs). MDs were used when outcomes were measured on the same scale, whereas SMDs were used when outcomes were measured using different scales. Considering the possible heterogeneity among studies, a random-effects model was used rather than a fixed-effects model. (8)For FT3, FT4, TRAb, TPOAb, and TgAb, positive MD values indicated a greater reduction from baseline in the TCM+MMI group than in the MMI-only group. For TSH, positive MD values indicated a greater increase or normalization toward the reference range in the TCM+MMI group. SUCRA rankings were interpreted according to these predefined beneficial directions.

Network meta-analysis was performed using Stata 15.1 software with a random-effects model, in accordance with PRISMA-NMA guidelines. This NMA included 15 intervention nodes: 14 TCM+MMI regimens and 1 MMI monotherapy control. Node-splitting analysis was used to assess consistency between direct and indirect comparisons. A P value > 0.05 was considered to indicate no statistically significant inconsistency. Stata software was used to graph the network relationships among the intervention programs: each node in the graph represents an intervention or control measure, and the connecting lines between the nodes represent direct head-to-head comparisons among the intervention programs; both node size and line width were positively correlated with the number of included studies (9, 10).

Intervention regimens were ranked using SUCRA values, which quantify the cumulative probability that each intervention is among the most effective treatments (11). SUCRA values range from 0 to 1, with values closer to 1 indicating a higher probability of being ranked among the most effective regimens and values closer to 0 indicating a lower probability. SUCRA values can be converted to percentages to reflect the relative ranking probability of each intervention. However, SUCRA rankings should be interpreted in the context of the corresponding effect estimates and their uncertainty, and should not be regarded as definitive evidence of clinical superiority. To test for possible publication bias resulting from small-sample studies, network funnel plots were drawn and visually assessed by symmetry tests (12).

## 3 Results

### 3.1 Results of literature search and screening

A total of 307 articles were retrieved from electronic databases, and no additional studies were identified through manual searching. After removing duplicates, 169 records remained for title and abstract screening, leading to the exclusion of 81 studies. The full texts of the remaining 88 studies were then reviewed, with further exclusions applied for reasons such as non-randomized design, incomplete data, conference abstracts, or inconsistency with the intervention criteria. Ultimately, 15 studies met the inclusion criteria and were included in the final analysis.(SF 1). (13–27) (Study characteristics are detailed in **Table 2**). A total of 1317 GD patients were included, with 663 in the TCM+MMI group and 654 in the MMI monotherapy group.

**Table 2:** Study characteristics included in network meta-analysis.

Author	Country	Year	Population	Age (mean+SD)	Total/male/female	Intervention	Control	Outcome
Zhao Yijing	China	2017	Graves’ disease	T:37.73 ± 9.17 C:34.35 ± 11.51	T:30/7/23 C:31/6/25	Xiehuo Xiaoying Formula combined with MMI Length of Intervention: 12 weeks	Methimazole Length of Intervention:12 weeks Freq:10-30mg/d	FT3,FT4,TSH,TRAb
Zhao Jianhong	China	2016	Graves’ disease	T:47.9 ± 6.6 C:46.9 ± 4.8	T:59/33/26 C:59/31/28	Xiaoyao Powder combined with MMI Length of Intervention: Half a year Freq: Daily dose	Methimazole Length of Intervention: Half a year Freq:30mg/d	FT3,FT4,TSH
Xia Simeng	China	2023	Graves’ disease	T:35.40 ± 11.43 C:35.37 ± 9.42	T:30/10/20 C:30/8/22	Modified Huagan Decoction combined with MMI Length of Intervention:8 weeks Freq: Take one dose daily, taking it before lunch and dinner.	Methimazole Length of Intervention:2months	FT3,FT4;TSH,TRAb
Xia Simeng	China	2024	Graves’ disease	T:34.82 ± 10.84 C:34.97 ± 9.32	T:28/10/18 C:29/9/20	Yueju Pill combined with MMI in conjunction with basic treatment Length of Intervention:8 weeks Freq: Each dose is 200ml. Take one dose per day, and take it twice (one hour before lunch and dinner).	Methimazole combined with basic treatment Length of Intervention:8 weeks	FT3,FT4,TSH,TRAb
Wang Xiaoqing	China	2018	Graves’ disease	T:37.45 ± 12.312 C:37.93 ± 12.574	T:40/14/26 C:40/11/29	Shugan Xiajia Formula combined with MMI Length of Intervention:4 weeks Freq: One dose per day, twice a day	Methimazole Length of Intervention:4 weeks Freq:10mg each time, twice a day	FT3,FT4,TSH
Tian Zhongyu	China	2020	Graves’ disease	T:34.02 ± 5.24 C:32.9 ± 4.88	T:45/10/35 C:45/8/37	Sanjie Xiaoying Decoction combined with MMI in conjunction with basic treatment Length of Intervention:12 weeks Freq: Take one dose daily, taking it twice a day in the morning and evening, and warm it up before consumption.	Methimazole combined with basic treatment Length of Intervention:12 weeks Freq:10mg per dose, 3 times a day	FT3,FT4,TSH,TRAb
Shi Ling	China	2017	Graves’ disease	NA	NA	Ziyin Xiaoying Formula combined with MMI in conjunction with basic treatment Length of Intervention:12 weeks Freq: Take 50ml each time, twice a day orally.	Methimazole combined with basic treatment Length of Intervention:12 weeks Freq:10-20mg,Twice daily	FT3,FT4,TSH
Jia Lin	China	2018	Graves’ disease	T:33.40 ± 7.44 C:32.93 ± 8.02	T:30/5/25 C:30/6/24	Longmu Xiaoyao Powder Granules combined with MMI in conjunction with basic treatment Length of Intervention:8 weeks Freq: One dose per day, divided in morning and evening	Methimazole combined with basic treatment Length of Intervention:8 weeks Freq:10mg	FT3,FT4,TSH,TRAb
Ji Zhihui	China	2018	Graves’ disease	T:36.34 ± 4.84 C:36.29 ± 4.82	T:50/13/37 C:50/11/39	Huotan Jiangni Formula combined with MMI Length of Intervention:3 months Freq: One dose per day, divided in morning and evening	Methimazole Length of Intervention:3 months Freq:30mg/d	FT3,FT4,TSH,TGAb,TPOAb
Duan Shanshan	China	2020	Graves’ disease	T: 42.46 ± 5.58 C: 43.09 ± 5.42	T:39/15/24 C:38/14/24	Modified Xiaoyao Powder combined with MMI in conjunction with basic treatment Length of Intervention:12 weeks Freq: Take 100ml each time 30 minutes after breakfast and dinner every day.	Methimazole combined with basic treatment Length of Intervention:12 weeks Freq:30mg/d	FT3,FT4,TSH,TRAb,TPOAb,
Dai Zhengqian	China	2020	Graves’ disease	T:39.2 ± 11.4 C:36.6 ± 10.2	T:56/12/44 C:117/32/85	Astragalus membranaceus Preparations combined with MMI Length of Intervention:6 months	Methimazole Length of Intervention:6 months Freq:10mg/d	FT4,TSH,TRAb
Liu Tianyi	China	2023	Graves’ disease	T:31.42 ± 11.64 C:34.66 ± 12.98	T:31/6/25 C:32/6/26	Chaihu Shaoyao Decoction combined with MMI Length of Intervention:12 weeks months Freq: Three times a day	Methimazole Length of Intervention:12 weeks Freq:30-45mg/d	FT3,FT4,TSH
Yang Hua	China	2017	Graves’ disease	T:42.92 ± 13.467 C:40.32 ± 14.192	T:65/8/57 C:65/17/48	Yingliu mixture combined with MMI in conjunction with basic treatment Length of Intervention:90 days Freq: Three times a day	Methimazole combined with basic treatment Length of Intervention:90 days Freq:5-25mg/d	FT3,FT4,TSH,TGAb,TPOAb,TRAb
Yang Hua	China	2015	Graves’ disease	T:36.50 ± 17.00 C:38.00 ± 24.50	T:40/8/32 C:40/11/29	Yingliu mixtur combined with MMI Length of Intervention:12 weeks months Freq: Three times a day	Methimazole Length of Intervention:12 weeks Freq:5-25mg/d	FT3,FT4,TSH,TGAb,TPOAb,TRAb
Huang Wenbin	China	2024	Graves’ disease	NA	T:54/12/42 C:54/11/43	HYD combined with MMI in conjunction with basic treatment Length of Intervention:12 weeeks Freq: Twice a day	Methimazole combined with basic treatment Length of Intervention:12 weeks	FT3,FT4,TSH,TRAb

MMI, Methimazole; T, experimental group; C, control group; NA, unavailable; Freq, frequency.

**Table 3:** Top 3 regimens for each outcome based on SUCRA.

Outcome	Rank 1 (SUCRA)	Rank 2 (SUCRA)	Rank 3 (SUCRA)
FT3	MHD+MMI (93.4%)	YJP+MMI (93.0%)	LMXP+MMI (90.2%)
FT4	MXYP+MMI (97.3%)	SXD+MMI (89.1%)	XYP+MMI (72.5%)
TSH	XYP+MMI (96.3%)	YJP+MMI (90.0%)	MHD+MMI (85.8%)
TRAb	MHD+MMI (88.2%)	YJP+MMI (87.0%)	AMP+MMI (73.3%)
TPOAb	HJF+MMI (100%)	MXYP+MMI (58.8%)	—
TgAb	No significant difference	—	—

### 3.2 Quality assessment of included studies

A total of 15 randomized controlled trials involving 1317 patients with GD were included in this study, evaluating 14 intervention protocols, Xiehuo Xiaoying Formula, Xiaoyao Powder, Modified Huagan Decoction, Yueju Pill, Shugan Xiajia Formula, Sanjie Xiaoying Decoction, Ziyin Xiaoying Formula, Longmu Xiaoyao Powder, Huotan Jiangni Formula, Modified Xiaoyao Powder, Astragalus membranaceus Preparations, Chaihu Shaoyao Decoction, Yingliu mixture, and HaiZao YuHu Decoction. FT3 and TSH levels were reported in 14 studies, FT4 levels in 15 studies, TRAb levels in 10 studies, TPOAb in 4 studies and TGAb in 3 studies. **Table 2** provides an overview of the characteristics of the included studies. SF 2 presents the risk of bias assessment for the included RCTs. All 15 included RCTs were conducted in China (single-center design for all), with sample sizes ranging from 59 to 173 patients per study (median = 60). ROB assessment (SF 2) showed that all studies had high risk only in the domain of performance bias due to unavoidable unblinded design; other domains were rated as low or unclear risk. According to comprehensive Cochrane evaluation criteria, all included studies were classified as moderate overall ROB rather than being divided into moderate and high ROB subgroups. Accordingly, the reliability of FT3, FT4, TSH, and TRAb was moderate, while that of TPOAb and TgAb was low. The primary source of performance bias was lack of blinding of participants/personnel, which is objectively unavoidable for oral TCM interventions due to distinct differences in taste, smell, and appearance between TCM formulations and MMI. No studies reported sample size calculation or trial registration (excluding the PROSPERO registration of this systematic review).

Transitivity assessment confirmed no critical heterogeneity across included studies: all studies used the same GD diagnostic criteria, consistent core intervention (MMI) and basic treatment, and identical outcome indices with consistent detection methods. Transitivity assumptions for network meta-analysis were therefore satisfied, supporting valid direct and indirect comparisons. Consistency tests (node-splitting analysis) showed all P > 0.05 for all outcomes, indicating no significant inconsistency between direct and indirect evidence. Since no studies were judged as high overall ROB, sensitivity analysis by excluding high-ROB studies was not performed. All outcomes were objective laboratory biomarkers, which may reduce the influence of unblinded performance bias on outcome assessment; however, the absence of blinding should still be considered when interpreting the findings.

### 3.3 Effect of TCM combined with MMI on FT3 level

The evidence plot of network meta-analysis of FT3 is shown in SF 3A. Consistency tests were satisfied for all outcomes (all P > 0.05). Clinically, reducing FT3 rapidly relieves palpitations, heat intolerance, and tremors, the core symptoms of hyperthyroidism. The results of the network meta-analysis showed that TCM plus MMI regimens tended to show greater FT3 reduction than MMI monotherapy. However, statistically significant between-group differences were observed only for the following regimens: Modified Huagan Decoction combined with MMI (MD = 4.52, 95% CI: 3.32–5.72), Yueju Pill combined with MMI (MD = 4.48, 95% CI: 3.28–5.68), Longmu Xiaoyao Powder combined with MMI (MD = 4.18, 95% CI: 2.60–5.76), Modified Xiaoyao Powder combined MMI (MD = 2.38, 95% CI: 2.01–2.75), Xiaoyao Powder combined with MMI (MD = 1.83, 95% CI: 1.31–2.35), Ziyin Xiaoying Formula combined with MMI (MD = 1.67, 95% CI: 0.99–2.35), Huotan Jiangni Formula combined with MMI (MD = 1.64, 95% CI: 1.21–2.07), Sanjie Xiaoying Decoction combined with MMI (MD = 1.42, 95% CI: 1.24–1.60), Chaihu Shaoyao Decoction combined with MMI (MD = 0.51, 95% CI: 0.15–0.87). Regarding the ranking of relative efficacy in reducing FT3, the combination of Modified Huagan Decoction and MMI had the highest surface under the cumulative ranking curve (SUCRA) value (93.4%, SF 3B), ranking first among all intervention regimens (**Table 3**). Pairwise comparisons among intervention regimens are shown in **Table 4** (league table).

**Table 4:** Ranking of each treatment based on SUCRA values, and the league table for relative effects of all treatments pairs on FT3:Mean difference (MD, pmol/L).

_D_	_M_	_C_	_E_	_L_	_N_	_J_	_G_	_I_	_A_	_B_	_K_	_H_	_F_
D	0.04 (-1.66,1.74)	0.34 (-1.64,2.32)	2.14 (0.88,3.40)	2.69 (1.38,4.00)	2.85 (1.47,4.23)	2.88 (1.60,4.16)	3.10 (1.89,4.31)	3.97 (2.32,5.62)	4.01 (2.76,5.26)	4.07 (2.49,5.65)	4.54 (1.17,7.91)	4.19 (2.46,5.92)	4.52 (3.32,5.72)
-0.04 (-1.74,1.66)	M	0.30 (-1.69,2.29)	2.10 (0.84,3.36)	2.65 (1.34,3.96)	2.81 (1.42,4.20)	2.84 (1.56,4.12)	3.06 (1.84,4.28)	3.93 (2.28,5.58)	3.97 (2.71,5.23)	4.03 (2.45,5.61)	4.50 (1.13,7.87)	4.15 (2.42,5.88)	4.48 (3.28,5.68)
-0.34 (-2.32,1.64)	-0.30 (-2.29,1.69)	C	1.80 (0.18,3.42)	2.35 (0.69,4.01)	2.51 (0.79,4.23)	2.54 (0.90,4.18)	2.76 (1.17,4.35)	3.63 (1.69,5.57)	3.67 (2.05,5.29)	3.73 (1.85,5.61)	4.20 (0.67,7.73)	3.85 (1.84,5.86)	4.18 (2.60,5.76)
-2.14 (-3.40,-0.88)	-2.10 (-3.36,-0.84)	-1.80 (-3.42,-0.18)	E	0.55 (-0.09,1.19)	0.71 (-0.07,1.49)	0.74 (0.17,1.31)	0.96 (0.54,1.38)	1.83 (0.64,3.02)	1.87 (1.35,2.39)	1.93 (0.84,3.02)	2.40 (-0.77,5.57)	2.05 (0.75,3.35)	2.38 (2.01,2.75)
-2.69 (-4.00,-1.38)	-2.65 (-3.96,-1.34)	-2.35 (-4.01,-0.69)	-0.55 (-1.19,0.09)	L	0.16 (-0.70,1.02)	0.19 (-0.48,0.86)	0.41 (-0.14,0.96)	1.28 (0.04,2.52)	1.32 (0.69,1.95)	1.38 (0.24,2.52)	1.85 (-1.34,5.04)	1.50 (0.15,2.85)	1.83 (1.31,2.35)
-2.85 (-4.23,-1.47)	-2.81 (-4.20,-1.42)	-2.51 (-4.23,-0.79)	-0.71 (-1.49,0.07)	-0.16 (-1.02,0.70)	N	0.03 (-0.78,0.84)	0.25 (-0.46,0.96)	1.12 (-0.20,2.44)	1.16 (0.38,1.94)	1.22 (-0.01,2.45)	1.69 (-1.54,4.92)	1.34 (-0.08,2.76)	1.67 (0.99,2.35)
-2.88 (-4.16,-1.60)	-2.84 (-4.12,-1.56)	-2.54 (-4.18,-0.90)	-0.74 (-1.31,-0.17)	-0.19 (-0.86,0.48)	-0.03 (-0.84,0.78)	J	0.22 (-0.25,0.69)	1.09 (-0.12,2.30)	1.13 (0.57,1.69)	1.19 (0.08,2.30)	1.66 (-1.52,4.84)	1.31 (-0.01,2.63)	1.64 (1.21,2.07)
-3.10 (-4.31,-1.89)	-3.06 (-4.28,-1.84)	-2.76 (-4.35,-1.17)	-0.96 (-1.38,-0.54)	-0.41 (-0.96,0.14)	-0.25 (-0.96,0.46)	-0.22 (-0.69,0.25)	G	0.87 (-0.27,2.02)	0.91 (0.50,1.32)	0.97 (-0.07,2.01)	1.44 (-1.72,4.60)	1.09 (-0.17,2.35)	1.42 (1.24,1.60)
-3.97 (-5.62,-2.32)	-3.93 (-5.58,-2.28)	-3.63 (-5.57,-1.69)	-1.83 (-3.02,-0.64)	-1.28 (-2.52,-0.04)	-1.12 (-2.44,0.20)	-1.09 (-2.30,0.12)	-0.87 (-2.02,0.27)	I	0.04 (-1.15,1.22)	0.10 (-1.43,1.62)	0.57 (-2.78,3.92)	0.22 (-1.46,1.90)	0.55 (-0.58,1.68)
-4.01 (-5.26,-2.76)	-3.97 (-5.23,-2.71)	-3.67 (-5.29,-2.05)	-1.87 (-2.39,-1.35)	-1.32 (-1.95,-0.69)	-1.16 (-1.94,-0.38)	-1.13 (-1.69,-0.57)	-0.91 (-1.32,-0.50)	-0.04 (-1.22,1.15)	A	0.06 (-1.02,1.14)	0.53 (-2.64,3.70)	0.18 (-1.12,1.48)	0.51 (0.15,0.87)
-4.07 (-5.65,-2.49)	-4.03 (-5.61,-2.45)	-3.73 (-5.61,-1.85)	-1.93 (-3.02,-0.84)	-1.38 (-2.52,-0.24)	-1.22 (-2.45,0.01)	-1.19 (-2.30,-0.08)	-0.97 (-2.01,0.07)	-0.10 (-1.62,1.43)	-0.06 (-1.14,1.02)	B	0.47 (-2.84,3.78)	0.12 (-1.49,1.73)	0.45 (-0.57,1.47)
-4.54 (-7.91,-1.17)	-4.50 (-7.87,-1.13)	-4.20 (-7.73,-0.67)	-2.40 (-5.57,0.77)	-1.85 (-5.04,1.34)	-1.69 (-4.92,1.54)	-1.66 (-4.84,1.52)	-1.44 (-4.60,1.72)	-0.57 (-3.92,2.78)	-0.53 (-3.70,2.64)	-0.47 (-3.78,2.84)	K	-0.35 (-3.74,3.04)	-0.02 (-3.17,3.13)
-4.19 (-5.92,-2.46)	-4.15 (-5.88,-2.42)	-3.85 (-5.86,-1.84)	-2.05 (-3.35,-0.75)	-1.50 (-2.85,-0.15)	-1.34 (-2.76,0.08)	-1.31 (-2.63,0.01)	-1.09 (-2.35,0.17)	-0.22 (-1.90,1.46)	-0.18 (-1.48,1.12)	-0.12 (-1.73,1.49)	0.35 (-3.04,3.74)	H	0.33 (-0.92,1.58)
-4.52 (-5.72,-3.32)	-4.48 (-5.68,-3.28)	-4.18 (-5.76,-2.60)	-2.38 (-2.75,-2.01)	-1.83 (-2.35,-1.31)	-1.67 (-2.35,-0.99)	-1.64 (-2.07,-1.21)	-1.42 (-1.60,-1.24)	-0.55 (-1.68,0.58)	-0.51 (-0.87,-0.15)	-0.45 (-1.47,0.57)	0.02 (-3.13,3.17)	-0.33 (-1.58,0.92)	F

### 3.4 Effect of TCM combined with MMI on FT4 level

The evidence plot of network meta-analysis of FT4 is shown in SF 4A. Consistency tests were satisfied for all outcomes (all P > 0.05). FT4 normalization helps maintain metabolic homeostasis and lower the risk of long-term complications. The results of the network meta-analysis showed that TCM plus MMI regimens tended to show greater FT4 reduction than MMI monotherapy. However, statistically significant between-group differences were observed only for the following regimens: Modified Xiaoyao Powder combined with MMI (MD = 6.87, 95% CI: 5.98-7.76), Sanjie Xiaoying Decoction combined with MMI (MD = 6.02, 95% CI: 4.12-7.92), Xiaoyao Powder combined with MMI (MD = 4.61, 95% CI: 3.03-6.19), Modified Huagan Decoction combined with MMI (MD = 4.60, 95% CI: 2.42-6.78), Yueju Pill combined with MMI (MD = 4.22, 95% CI: 2.04-6.40), Ziyin Xiaoying Formula combined with MMI (MD = 4.11, 95% CI: 2.21-6.01), Longmu Xiaoyao Powder combined with MMI (MD = 4.06, 95% CI: 1.26-6.86), Huotan Jiangni Formula combined with MMI (MD = 3.54, 95% CI: 2.78-4.30), Astragalus-containing preparations combined with MMI (MD = 2.71, 95% CI: 1.02-4.40), Shugan Xiaoying Formula combined with MMI (MD = 2.20, 95% CI: 0.15-4.25). Regarding the ranking of relative efficacy in reducing FT4, the combination of Modified Xiaoyao Powder and MMI had the highest surface under the cumulative ranking curve (SUCRA) value (97.3%, SF4B), ranking first among all intervention regimens (**Table 3**). Pairwise comparisons among intervention regimens are shown in **Table 5** (league table).

**Table 5:** Ranking of each treatment based on SUCRA values, and the league table for relative effects of all treatments pairs on FT4:Mean difference (MD, pmol/L).

_F_	_H_	_M_	_E_	_N_	_O_	_D_	_K_	_A_	_I_	_C_	_J_	_L_	_B_	_G_
F	0.85 (-1.25,2.95)	2.26 (0.45,4.07)	2.27 (-0.09,4.63)	2.65 (0.30,5.00)	2.76 (0.66,4.86)	2.81 (-0.13,5.75)	3.33 (2.16,4.50)	4.16 (2.25,6.07)	4.67 (2.44,6.90)	5.41 (2.51,8.31)	5.40 (2.41,8.39)	8.11 (-2.28,18.50)	6.16 (4.55,7.77)	6.87 (5.98,7.76)
-0.85 (-2.95,1.25)	H	1.41 (-1.06,3.88)	1.42 (-1.47,4.31)	1.80 (-1.09,4.69)	1.91 (-0.78,4.60)	1.96 (-1.42,5.34)	2.48 (0.44,4.52)	3.31 (0.77,5.85)	3.82 (1.03,6.61)	4.56 (1.21,7.91)	4.55 (1.12,7.98)	7.26 (-3.27,17.79)	5.31 (2.99,7.63)	6.02 (4.12,7.92)
-2.26 (-4.07,-0.45)	-1.41 (-3.88,1.06)	M	0.01 (-2.68,2.70)	0.39 (-2.30,3.08)	0.50 (-1.97,2.97)	0.55 (-2.66,3.76)	1.07 (-0.68,2.82)	1.90 (-0.41,4.21)	2.41 (-0.17,4.99)	3.15 (-0.03,6.33)	3.14 (-0.12,6.41)	5.85 (-4.62,16.32)	3.90 (1.83,5.97)	4.61 (3.03,6.19)
-2.27 (-4.63,0.09)	-1.42 (-4.31,1.47)	-0.01 (-2.70,2.68)	E	0.38 (-2.70,3.46)	0.49 (-2.41,3.39)	0.54 (-3.01,4.09)	1.06 (-1.25,3.37)	1.89 (-0.87,4.65)	2.40 (-0.59,5.39)	3.14 (-0.38,6.66)	3.13 (-0.47,6.73)	5.84 (-4.74,16.42)	3.89 (1.33,6.45)	4.60 (2.42,6.78)
-2.65 (-5.00,-0.30)	-1.80 (-4.69,1.09)	-0.39 (-3.08,2.30)	-0.38 (-3.46,2.70)	N	0.11 (-2.78,3.00)	0.16 (-3.39,3.71)	0.68 (-1.62,2.98)	1.51 (-1.24,4.26)	2.02 (-0.97,5.01)	2.76 (-0.76,6.28)	2.75 (-0.84,6.34)	5.46 (-5.12,16.04)	3.51 (0.96,6.06)	4.22 (2.04,6.40)
-2.76 (-4.86,-0.66)	-1.91 (-4.60,0.78)	-0.50 (-2.97,1.97)	-0.49 (-3.39,2.41)	-0.11 (-3.00,2.78)	O	0.05 (-3.34,3.44)	0.57 (-1.48,2.62)	1.40 (-1.14,3.94)	1.91 (-0.89,4.71)	2.65 (-0.70,6.00)	2.64 (-0.79,6.07)	5.35 (-5.18,15.88)	3.40 (1.07,5.73)	4.11 (2.21,6.01)
-2.81 (-5.75,0.13)	-1.96 (-5.34,1.42)	-0.55 (-3.76,2.66)	-0.54 (-4.09,3.01)	-0.16 (-3.71,3.39)	-0.05 (-3.44,3.34)	D	0.52 (-2.38,3.42)	1.35 (-1.92,4.62)	1.86 (-1.61,5.33)	2.60 (-1.33,6.53)	2.59 (-1.41,6.59)	5.30 (-5.42,16.02)	3.35 (0.25,6.45)	4.06 (1.26,6.86)
-3.33 (-4.50,-2.16)	-2.48 (-4.52,-0.44)	-1.07 (-2.82,0.68)	-1.06 (-3.37,1.25)	-0.68 (-2.98,1.62)	-0.57 (-2.62,1.48)	-0.52 (-3.42,2.38)	K	0.83 (-1.02,2.68)	1.34 (-0.84,3.52)	2.08 (-0.78,4.94)	2.07 (-0.88,5.03)	4.78 (-5.60,15.16)	2.83 (1.29,4.37)	3.54 (2.78,4.30)
-4.16 (-6.07,-2.25)	-3.31 (-5.85,-0.77)	-1.90 (-4.21,0.41)	-1.89 (-4.65,0.87)	-1.51 (-4.26,1.24)	-1.40 (-3.94,1.14)	-1.35 (-4.62,1.92)	-0.83 (-2.68,1.02)	A	0.51 (-2.15,3.17)	1.25 (-1.99,4.49)	1.24 (-2.08,4.56)	3.95 (-6.54,14.44)	2.00 (-0.16,4.16)	2.71 (1.02,4.40)
-4.67 (-6.90,-2.44)	-3.82 (-6.61,-1.03)	-2.41 (-4.99,0.17)	-2.40 (-5.39,0.59)	-2.02 (-5.01,0.97)	-1.91 (-4.71,0.89)	-1.86 (-5.33,1.61)	-1.34 (-3.52,0.84)	-0.51 (-3.17,2.15)	I	0.74 (-2.70,4.18)	0.73 (-2.79,4.25)	3.44 (-7.11,13.99)	1.49 (-0.96,3.94)	2.20 (0.15,4.25)
-5.41 (-8.31,-2.51)	-4.56 (-7.91,-1.21)	-3.15 (-6.33,0.03)	-3.14 (-6.66,0.38)	-2.76 (-6.28,0.76)	-2.65 (-6.00,0.70)	-2.60 (-6.53,1.33)	-2.08 (-4.94,0.78)	-1.25 (-4.49,1.99)	-0.74 (-4.18,2.70)	C	-0.01 (-3.98,3.97)	2.70 (-8.01,13.41)	0.75 (-2.32,3.82)	1.46 (-1.30,4.22)
-5.40 (-8.39,-2.41)	-4.55 (-7.98,-1.12)	-3.14 (-6.41,0.12)	-3.13 (-6.73,0.47)	-2.75 (-6.34,0.84)	-2.64 (-6.07,0.79)	-2.59 (-6.59,1.41)	-2.07 (-5.03,0.88)	-1.24 (-4.56,2.08)	-0.73 (-4.25,2.79)	0.01 (-3.97,3.98)	J	2.71 (-8.03,13.45)	0.76 (-2.40,3.91)	1.47 (-1.39,4.33)
-8.11 (-18.50,2.28)	-7.26 (-17.79,3.27)	-5.85 (-16.32,4.62)	-5.84 (-16.42,4.74)	-5.46 (-16.04,5.12)	-5.35 (-15.88,5.18)	-5.30 (-16.02,5.42)	-4.78 (-15.16,5.60)	-3.95 (-14.44,6.54)	-3.44 (-13.99,7.11)	-2.70 (-13.41,8.01)	-2.71 (-13.45,8.03)	L	-1.95 (-12.39,8.49)	-1.24 (-11.59,9.11)
-6.16 (-7.77,-4.55)	-5.31 (-7.63,-2.99)	-3.90 (-5.97,-1.83)	-3.89 (-6.45,-1.33)	-3.51 (-6.06,-0.96)	-3.40 (-5.73,-1.07)	-3.35 (-6.45,-0.25)	-2.83 (-4.37,-1.29)	-2.00 (-4.16,0.16)	-1.49 (-3.94,0.96)	-0.75 (-3.82,2.32)	-0.76 (-3.91,2.40)	1.95 (-8.49,12.39)	B	0.71 (-0.63,2.05)
-6.87 (-7.76,-5.98)	-6.02 (-7.92,-4.12)	-4.61 (-6.19,-3.03)	-4.60 (-6.78,-2.42)	-4.22 (-6.40,-2.04)	-4.11 (-6.01,-2.21)	-4.06 (-6.86,-1.26)	-3.54 (-4.30,-2.78)	-2.71 (-4.40,-1.02)	-2.20 (-4.25,-0.15)	-1.46 (-4.22,1.30)	-1.47 (-4.33,1.39)	1.24 (-9.11,11.59)	-0.71 (-2.05,0.63)	G

Positive MD values indicate a greater reduction from baseline in the TCM+MMI group than in the MMI-only group.

### 3.5 Effect of TCM combined with MMI on TSH levels

The evidence plot of network meta-analysis of TSH is shown in SF5A. Consistency tests were satisfied for all outcomes (all P > 0.05). TSH recovery indicates restoration of the hypothalamic-pituitary-thyroid axis function. Findings of the network meta-analysis revealed that TCM plus MMI regimens tended to show greater TSH recovery than MMI monotherapy. However, only the following regimens showed statistically significant intergroup differences: Xiaoyao Powder combined with MMI (MD = 2.18, 95% CI: 1.85-2.51), Yueju Pill combined with MMI (MD = 1.98, 95% CI: 1.65-2.31), Modified Huagan Decoction combined with MMI (MD = 1.85, 95% CI: 1.49-2.21), Xiehuo Xiaoying Formula combined with MMI (MD = 0.97, 95% CI: 0.95-0.99), Huotan Jiangni Formula combined with MMI (MD = 0.55, 95% CI: 0.41-0.69), Modified Xiaoyao Powder combined with MMI (MD = 0.41, 95% CI: 0.37-0.45), Sanjie Xiaoying Decoction combined with MMI (MD = 0.34, 95% CI: 0.06-0.62), Longmu Xiaoyao Powder combined with MMI (MD = 0.14, 95% CI: 0.03-0.26), Ziyin Xiaoying Formula combined with MMI (MD = 0.14, 95% CI: 0.03-0.25). Regarding the ranking of relative efficacy in TSH regulation, the combination of Xiaoyao Powder and MMI had the highest surface under the cumulative ranking curve (SUCRA) value (96.3%, SF5B), ranking first among all intervention regimens (**Table 3**). Pairwise comparisons among intervention regimens are shown in **Table 6**(league table). Because several TSH comparisons were derived from single small RCTs, the TSH estimates and SUCRA rankings should be interpreted cautiously.

**Table 6:** Ranking of each treatment based on SUCRA values, and the league table for relative effects of all treatments pairs on TSH: Mean difference (MD, mIU/L).

_M_	_N_	_E_	_L_	_A_	_K_	_I_	_F_	_H_	_B_	_C_	_D_	_O_	_J_	_G_
M	-0.20 (-0.67,0.27)	-0.33 (-0.82,0.16)	-1.21 (-1.54,-0.88)	-1.04 (-2.38,0.30)	-1.63 (-1.99,-1.27)	-1.60 (-3.53,0.33)	-1.77 (-2.11,-1.43)	-1.84 (-2.28,-1.40)	-1.85 (-2.43,-1.27)	-2.09 (-5.37,1.20)	-2.04 (-2.39,-1.68)	-2.04 (-2.39,-1.69)	-2.17 (-2.79,-1.55)	-2.18 (-2.51,-1.85)
0.20 (-0.27,0.67)	N	-0.13 (-0.62,0.36)	-1.01 (-1.34,-0.68)	-0.84 (-2.17,0.49)	-1.43 (-1.78,-1.08)	-1.40 (-3.33,0.53)	-1.57 (-1.90,-1.24)	-1.64 (-2.07,-1.21)	-1.65 (-2.22,-1.08)	-1.89 (-5.17,1.40)	-1.84 (-2.18,-1.49)	-1.84 (-2.19,-1.49)	-1.97 (-2.59,-1.36)	-1.98 (-2.31,-1.65)
0.33 (-0.16,0.82)	0.13 (-0.36,0.62)	E	-0.88 (-1.24,-0.52)	-0.71 (-2.05,0.63)	-1.30 (-1.68,-0.92)	-1.27 (-3.21,0.67)	-1.44 (-1.80,-1.08)	-1.51 (-1.97,-1.05)	-1.52 (-2.11,-0.93)	-1.76 (-5.04,1.53)	-1.71 (-2.08,-1.33)	-1.71 (-2.09,-1.33)	-1.84 (-2.48,-1.21)	-1.85 (-2.21,-1.49)
1.21 (0.88,1.54)	1.01 (0.68,1.34)	0.88 (0.52,1.24)	L	0.17 (-1.12,1.46)	-0.42 (-0.56,-0.28)	-0.39 (-2.29,1.51)	-0.56 (-0.61,-0.51)	-0.63 (-0.92,-0.35)	-0.64 (-1.11,-0.17)	-0.88 (-4.14,2.39)	-0.83 (-0.94,-0.71)	-0.83 (-0.94,-0.72)	-0.96 (-1.49,-0.44)	-0.97 (-0.99,-0.95)
1.04 (-0.30,2.38)	0.84 (-0.49,2.17)	0.71 (-0.63,2.05)	-0.17 (-1.46,1.12)	A	-0.59 (-1.89,0.71)	-0.56 (-2.86,1.74)	-0.73 (-2.02,0.56)	-0.80 (-2.12,0.52)	-0.81 (-2.19,0.57)	-1.05 (-4.56,2.47)	-1.00 (-2.29,0.30)	-1.00 (-2.30,0.30)	-1.13 (-2.53,0.26)	-1.14 (-2.43,0.15)
1.63 (1.27,1.99)	1.43 (1.08,1.78)	1.30 (0.92,1.68)	0.42 (0.28,0.56)	0.59 (-0.71,1.89)	K	0.03 (-1.88,1.94)	-0.14 (-0.28,0.00)	-0.21 (-0.53,0.10)	-0.22 (-0.71,0.27)	-0.46 (-3.73,2.81)	-0.41 (-0.58,-0.23)	-0.41 (-0.59,-0.23)	-0.54 (-1.08,-0.00)	-0.55 (-0.69,-0.41)
1.60 (-0.33,3.53)	1.40 (-0.53,3.33)	1.27 (-0.67,3.21)	0.39 (-1.51,2.29)	0.56 (-1.74,2.86)	-0.03 (-1.94,1.88)	I	-0.17 (-2.07,1.73)	-0.24 (-2.17,1.68)	-0.25 (-2.21,1.71)	-0.49 (-4.27,3.30)	-0.44 (-2.34,1.47)	-0.44 (-2.35,1.47)	-0.57 (-2.55,1.40)	-0.58 (-2.48,1.32)
1.77 (1.43,2.11)	1.57 (1.24,1.90)	1.44 (1.08,1.80)	0.56 (0.51,0.61)	0.73 (-0.56,2.02)	0.14 (-0.00,0.28)	0.17 (-1.73,2.07)	F	-0.07 (-0.36,0.22)	-0.08 (-0.55,0.39)	-0.32 (-3.58,2.95)	-0.27 (-0.39,-0.15)	-0.27 (-0.39,-0.15)	-0.40 (-0.93,0.12)	-0.41 (-0.45,-0.37)
1.84 (1.40,2.28)	1.64 (1.21,2.07)	1.51 (1.05,1.97)	0.63 (0.35,0.92)	0.80 (-0.52,2.12)	0.21 (-0.10,0.53)	0.24 (-1.68,2.17)	0.07 (-0.22,0.36)	H	-0.01 (-0.56,0.54)	-0.24 (-3.53,3.04)	-0.20 (-0.50,0.11)	-0.20 (-0.50,0.11)	-0.33 (-0.93,0.26)	-0.34 (-0.62,-0.06)
1.85 (1.27,2.43)	1.65 (1.08,2.22)	1.52 (0.93,2.11)	0.64 (0.17,1.11)	0.81 (-0.57,2.19)	0.22 (-0.27,0.71)	0.25 (-1.71,2.21)	0.08 (-0.39,0.55)	0.01 (-0.54,0.56)	B	-0.24 (-3.54,3.07)	-0.19 (-0.67,0.30)	-0.19 (-0.68,0.30)	-0.32 (-1.03,0.38)	-0.33 (-0.80,0.14)
2.09 (-1.20,5.37)	1.89 (-1.40,5.17)	1.76 (-1.53,5.04)	0.88 (-2.39,4.14)	1.05 (-2.47,4.56)	0.46 (-2.81,3.73)	0.49 (-3.30,4.27)	0.32 (-2.95,3.58)	0.24 (-3.04,3.53)	0.24 (-3.07,3.54)	C	0.05 (-3.22,3.32)	0.05 (-3.22,3.32)	-0.09 (-3.40,3.22)	-0.09 (-3.36,3.17)
2.04 (1.68,2.39)	1.84 (1.49,2.18)	1.71 (1.33,2.08)	0.83 (0.71,0.94)	1.00 (-0.30,2.29)	0.41 (0.23,0.58)	0.44 (-1.47,2.34)	0.27 (0.15,0.39)	0.20 (-0.11,0.50)	0.19 (-0.30,0.67)	-0.05 (-3.32,3.22)	D	-0.00 (-0.16,0.16)	-0.14 (-0.67,0.40)	-0.14 (-0.26,-0.03)
2.04 (1.69,2.39)	1.84 (1.49,2.19)	1.71 (1.33,2.09)	0.83 (0.72,0.94)	1.00 (-0.30,2.30)	0.41 (0.23,0.59)	0.44 (-1.47,2.35)	0.27 (0.15,0.39)	0.20 (-0.11,0.50)	0.19 (-0.30,0.68)	-0.05 (-3.32,3.22)	0.00 (-0.16,0.16)	O	-0.13 (-0.67,0.40)	-0.14 (-0.25,-0.03)
2.17 (1.55,2.79)	1.97 (1.36,2.59)	1.84 (1.21,2.48)	0.96 (0.44,1.49)	1.13 (-0.26,2.53)	0.54 (0.00,1.08)	0.57 (-1.40,2.55)	0.40 (-0.12,0.93)	0.33 (-0.26,0.93)	0.32 (-0.38,1.03)	0.09 (-3.22,3.40)	0.14 (-0.40,0.67)	0.13 (-0.40,0.67)	J	-0.01 (-0.53,0.51)
2.18 (1.85,2.51)	1.98 (1.65,2.31)	1.85 (1.49,2.21)	0.97 (0.95,0.99)	1.14 (-0.15,2.43)	0.55 (0.41,0.69)	0.58 (-1.32,2.48)	0.41 (0.37,0.45)	0.34 (0.06,0.62)	0.33 (-0.14,0.80)	0.09 (-3.17,3.36)	0.14 (0.03,0.26)	0.14 (0.03,0.25)	0.01 (-0.51,0.53)	G

Positive MD values indicate a greater increase or normalization of TSH in the TCM+MMI group than in the MMI-only group.

### 3.6 Effect of TCM combined with MMI on TgAb levels

The evidence plot of network meta-analysis of TgAb is shown in SF6A. The results of consistency test for direct versus indirect comparisons showed that all P-values were >0.05, suggesting inter-study consistency. The results of the network meta-analysis showed that TCM plus MMI regimens had a non-significant trend toward greater TgAb reduction than MMI monotherapy, but none of the regimens achieved statistical significance. This represents an important limitation of the present study, indicating that the beneficial effect of TCM combined with MMI on TgAb could not be statistically confirmed despite the observed favorable trend. Pairwise comparisons among intervention regimens are shown in **Table 7** (league table).

**Table 7:** Ranking of each treatment based on SUCRA values, and the league table for relative effects of all treatments pairs on TgAb: Mean difference (MD, IU/mL).

_A_	_B_	_C_
A	7.79 (-140.75,156.32)	66.32 (-161.12,293.75)
-7.79 (-156.32,140.75)	B	58.53 (-119.36,236.41)
-66.32 (-293.75,161.12)	-58.53 (-236.41,119.36)	C

### 3.7 Effect of TCM Combined with MMI on TPOAb Levels

The evidence network plot of the network meta-analysis for TPOAb is shown in SF7A. Results of the consistency test for direct and indirect comparisons indicated that all P-values were > 0.05, suggesting consistency across studies. The results of the network meta-analysis showed that TCM plus MMI regimens tended to be superior to MMI monotherapy in regulating TPOAb. However, only the following two regimens showed statistically significant intergroup differences: Huotan Jiangni Formula combined with MMI (MD = 75.55, 95% CI: 68.79-82.31), Modified Xiaoyao Powder combined with MMI (MD = 18.53, 95% CI: 16.81-20.25). Regarding the relative efficacy ranking for TPOAb regulation, Huotan Jiangni Formula combined with MMI had the highest surface under the cumulative ranking curve (SUCRA) value (100%, see SF7B), ranking first among the included regimens (**Table 3**). Pairwise comparisons among intervention regimens are shown in **Table 8** (league table).

**Table 8:** Ranking of each treatment based on SUCRA values, and the league table for relative effects of all treatments pairs on TPOAb: Mean difference (MD, IU/mL).

_D_	_A_	_C_	_B_
D	57.02 (50.05,63.99)	72.71 (27.89,117.53)	75.55 (68.79,82.31)
-57.02 (-63.99,-50.05)	A	15.69 (-28.65,60.03)	18.53 (16.81,20.25)
-72.71 (-117.53,-27.89)	-15.69 (-60.03,28.65)	C	2.84 (-41.46,47.14)
-75.55 (-82.31,-68.79)	-18.53 (-20.25,-16.81)	-2.84 (-47.14,41.46)	B

### 3.8 Effect of TCM combined with MMI on TRAb levels

The evidence network plot of network meta-analysis for thyrotropin receptor antibody (TRAb) is shown in SF8A. Results of the consistency test for direct and indirect comparisons showed that all P-values were > 0.05, indicating consistency across studies. Findings from the network meta-analysis suggested that TCM plus MMI regimens tended to show greater TRAb reduction than MMI monotherapy. However, only the following regimens exhibited statistically significant intergroup differences: Modified Huagan Decoction combined with MMI (MD = 5.27, 95% CI: 0.96-9.58), Yueju Pill combined with MMI (MD = 5.19, 95% CI: 0.78-9.60), Astragalus-containing preparations combined with MMI (MD = 3.15, 95% CI: 1.33-4.97), Modified Xiaoyao Powder combined with MMI (MD = 1.83, 95% CI: 1.35-2.31), Sanjie Xiaoying Decoction combined with MMI (MD = 0.86, 95% CI: 0.26-1.46). Regarding the relative efficacy ranking for TRAb regulation, the combination of Modified Huagan Decoction and MMI had the highest surface under the cumulative ranking curve (SUCRA) value (88.2%, see SF8B), ranking first (**Table 3**). It showed the highest SUCRA ranking for TRAb reduction among the included regimens. Pairwise comparisons among intervention regimens are shown in **Table 9** (league table).

**Table 9:** Ranking of each treatment based on SUCRA values, and the league table for relative effects of all treatments pairs on TRAb: Mean difference (MD, IU/mL).

_D_	_J_	_A_	_H_	_E_	_I_	_B_	_G_	_C_	_F_
D	0.08 (-6.09,6.25)	2.12 (-2.56,6.80)	2.70 (-2.59,7.98)	3.44 (-0.90,7.78)	3.90 (-1.62,9.42)	3.94 (-0.95,8.83)	4.41 (0.06,8.76)	5.01 (0.32,9.70)	5.27 (0.96,9.58)
-0.08 (-6.25,6.09)	J	2.04 (-2.73,6.81)	2.62 (-2.75,7.99)	3.36 (-1.08,7.80)	3.82 (-1.79,9.43)	3.86 (-1.12,8.84)	4.33 (-0.13,8.79)	4.93 (0.15,9.71)	5.19 (0.78,9.60)
-2.12 (-6.80,2.56)	-2.04 (-6.81,2.73)	A	0.58 (-2.98,4.13)	1.32 (-0.56,3.20)	1.78 (-2.12,5.68)	1.82 (-1.11,4.75)	2.29 (0.38,4.20)	2.89 (0.31,5.47)	3.15 (1.33,4.97)
-2.70 (-7.98,2.59)	-2.62 (-7.99,2.75)	-0.58 (-4.13,2.98)	H	0.74 (-2.35,3.84)	1.20 (-3.41,5.82)	1.24 (-2.58,5.07)	1.71 (-1.40,4.83)	2.31 (-1.25,5.88)	2.57 (-0.48,5.63)
-3.44 (-7.78,0.90)	-3.36 (-7.80,1.08)	-1.32 (-3.20,0.56)	-0.74 (-3.84,2.35)	E	0.46 (-3.03,3.95)	0.50 (-1.85,2.85)	0.97 (0.20,1.74)	1.57 (-0.33,3.47)	1.83 (1.35,2.31)
-3.90 (-9.42,1.62)	-3.82 (-9.43,1.79)	-1.78 (-5.68,2.12)	-1.20 (-5.82,3.41)	-0.46 (-3.95,3.03)	I	0.04 (-4.11,4.19)	0.51 (-3.00,4.02)	1.11 (-2.80,5.02)	1.37 (-2.08,4.82)
-3.94 (-8.83,0.95)	-3.86 (-8.84,1.12)	-1.82 (-4.75,1.11)	-1.24 (-5.07,2.58)	-0.50 (-2.85,1.85)	-0.04 (-4.19,4.11)	B	0.47 (-1.91,2.85)	1.07 (-1.87,4.01)	1.33 (-0.97,3.63)
-4.41 (-8.76,-0.06)	-4.33 (-8.79,0.13)	-2.29 (-4.20,-0.38)	-1.71 (-4.83,1.40)	-0.97 (-1.74,-0.20)	-0.51 (-4.02,3.00)	-0.47 (-2.85,1.91)	G	0.60 (-1.33,2.53)	0.86 (0.26,1.46)
-5.01 (-9.70,-0.32)	-4.93 (-9.71,-0.15)	-2.89 (-5.47,-0.31)	-2.31 (-5.88,1.25)	-1.57 (-3.47,0.33)	-1.11 (-5.02,2.80)	-1.07 (-4.01,1.87)	-0.60 (-2.53,1.33)	C	0.26 (-1.58,2.10)
-5.27 (-9.58,-0.96)	-5.19 (-9.60,-0.78)	-3.15 (-4.97,-1.33)	-2.57 (-5.63,0.48)	-1.83 (-2.31,-1.35)	-1.37 (-4.82,2.08)	-1.33 (-3.63,0.97)	-0.86 (-1.46,-0.26)	-0.26 (-2.10,1.58)	F

**Table 10:** Structured adverse-event reporting of the included randomized controlled trials.

Study	Regimen	Sample size T/C	AE reporting status	Liver dysfunction T/C	Leukopenia T/C	Skin/allergic reactions T/C	Gastrointestinal symptoms T/C	Other reported AE	Severe AE	Withdrawal due to AE
Zhao Yijing, 2017	Xiehuo Xiaoying Formula + MMI	T30/C31	Reported	1/4	0/0	2/2	0/0	NR	NR	NR
Zhao Jianhong, 2016	Xiaoyao Powder + MMI	T59/C59	Reported	1/3	1/3	2/3	0/0	Renal function injury 0/4; hypothyroidism 5/9	NR	NR
Xia Simeng, 2023	Modified Huagan Decoction + MMI	T30/C30	Reported	1/2	0/3	0/0	0/0	NR	NR	NR
Xia Simeng, 2024	Yueju Pill + MMI	T28/C29	Reported	0/0	0/0	0/0	2/0	Loose stools 1/0; stomach cold/reduced appetite 1/0	No severe AE reported	0/0
Wang Xiaoqing, 2018	Shugan Xiajia Formula + MMI	T40/C40	Explicitly no AE	0/0	0/0	0/0	0/0	No rash/itching; blood routine and liver function normal	0/0	0/0
Tian Zhongyu, 2020	Sanjie Xiaoying Decoction + MMI	T45/C45	Not reported	NR	NR	NR	NR	NR	NR	NR
Shi Ling, 2017	Ziyin Xiaoying Formula + MMI	T30/C30	Explicitly no AE	0/0	0/0	0/0	0/0	Safety indicators normal	0/0	0/0
Jia Lin, 2018	Longmu Xiaoyao Powder + MMI	T30/C30	Event described; relationship unclear	0/0	0/0	0/0	1/0	Gastric distension/discomfort, considered related to cold food; resolved after rest	0/0	0/0
Ji Zhihui, 2018	Huotan Jiangni Formula + MMI	T50/C50	Not reported	NR	NR	NR	NR	NR	NR	NR
Duan Shanshan, 2023	Modified Xiaoyao Powder + MMI	T39/C38	Explicitly no AE	0/0	0/0	0/0	0/0	No abnormalities in liver/kidney function, blood routine, myocardial enzymes, heart rate, pulse or blood pressure	0/0	0/0
Dai Zhengqian, 2020	Astragalus preparations + MMI	T56/C117	Not reported	NR	NR	NR	NR	NR	NR	NR
Liu Tianyi, 2023	Chaihu Shaoyao Decoction + MMI	T31/C32	Reported	0/0	0/0	0/1	0/0	Control group: rash 1 case; treatment group: 2 dropouts due to poor compliance	0/0	0/1
Yang Hua, 2017	Yingliu mixture + MMI	T65/C65	Safety monitored but AE outcomes not reported	NR	NR	NR	NR	NR	NR	NR
Yang Hua, 2015	Yingliu mixture + MMI	T40/C40	Reported	5/1	5/11	NR	NR	Safety grades: T L1=31, L2=2, L3=7, L4=0; C L1=28, L2=4, L3=8, L4=0	0/0 level 4	0/0
Huang Wenbin, 2024	Haizao Yuhu Decoction + MMI	T54/C54	Reported	2/1	2/1	0/3	1/0	Other discomfort 2/1: GI discomfort and mild limb discomfort in T; nocturnal lower-limb distension/pain in C	0/0	0/0

AE, adverse event; T, TCM plus MMI group; C, MMI-only control group; NR, not reported. Studies without explicit adverse-event information were coded as “NR” rather than “no adverse events.” Studies explicitly reporting no adverse events or normal safety indicators were coded as “explicitly no AE.” Because AE definitions, monitoring frequency, severity grading, and reporting methods varied across studies, these data were summarized descriptively and were not included in quantitative network meta-analysis.

### 3.9 Adverse reactions

Adverse-event reporting was incomplete and heterogeneous across the included studies. Therefore, AE data were summarized descriptively and are presented in **Table 10**. Studies without explicit AE information were coded as “not reported” rather than “no adverse events,” whereas studies explicitly reporting no AEs or normal safety indicators were coded as “explicitly no AE.” Across the included RCTs, reported AEs mainly included liver dysfunction, leukopenia, skin or allergic reactions, gastrointestinal symptoms, and other mild discomfort. No severe AEs were explicitly reported in the available data. However, because AE definitions, monitoring frequency, severity grading, and reporting methods varied across studies, quantitative network meta-analysis of AEs was not performed. Therefore, the current evidence is insufficient to conclude that TCM+MMI is safer than MMI monotherapy.

### 3.10 Funnel diagram characteristics

To detect publication bias, funnel plots were drawn separately for each outcome indicator. Visual assessment showed no obvious funnel plot asymmetry. Quantitative tests (e.g., Egger’s test) were not performed due to the small number of included studies (n=15). Therefore, publication bias could not be formally excluded. These findings suggest no obvious small-study effects based on visual assessment alone.

## 4 Discussion

This study performed a network meta-analysis of 15 RCTs, including 1,317 patients with GD and 14 distinct TCM+MMI combination regimens. The results suggested that TCM combined with MMI may have relative advantages over MMI monotherapy in improving thyroid function (as reflected by FT3, FT4, and TSH levels) and reducing selected autoantibody titers, particularly TRAb and TPOAb. In addition, AE data were summarized descriptively because AE reporting was incomplete and heterogeneous across the included RCTs. The available evidence is insufficient to determine whether TCM+MMI reduces adverse events compared with MMI monotherapy. Notably, the certainty of the above findings is limited by the methodological shortcomings of the included studies: all RCTs were single-center with small sample sizes, all were judged as having moderate overall risk of bias, and no studies reported long-term follow-up data. SUCRA rankings used in this study reflect relative comparative efficacy within the included study population and cannot be interpreted as definitive clinical superiority; these rankings require further validation in high-quality trials.

In the regulation of thyroid function, different integrated traditional Chinese and Western medicine regimens showed different relative rankings for thyroid hormone-related outcomes in the present network meta-analysis. The following mechanistic explanations are tentative based on TCM theory, preclinical pharmacological studies and existing literature, and are not directly supported by the clinical data of this study, requiring further experimental and clinical validation (28).

Regarding thyroid function regulation, different integrated traditional Chinese and Western medicine regimens showed different relative rankings for thyroid hormone-related outcomes in the present network meta-analysis.

Modified Huagan Decoction (MHD) plus methimazole (MMI) showed the highest SUCRA ranking for FT3 reduction; Modified Xiaoyao Powder (MXYP) plus MMI showed the highest SUCRA ranking for FT4 reduction; and Xiaoyao Powder (XYP) plus MMI showed the highest SUCRA ranking for TSH regulation. These findings suggest potential short-term advantages in thyroid-function regulation, but should not be interpreted as definitive mechanistic evidence. Modified Huagan Decoction originates from Jing Yue Quan Shu. Its core pathogenesis involves liver qi stagnation transforming into fire, hyperactive liver fire, and consumption of yin fluid. (24) Its therapeutic principles are soothing liver qi and resolving stagnation, clearing liver fire, nourishing yin and dissipating masses, regulating qi and resolving phlegm. In this formula: Paeoniae Radix Alba (Baishao) restrains yin, softens the liver, and absorbs floating yang; Moutan Cortex (Mudanpi) cools blood and nourishes yin; Prunellae Spica (Xiakucao) and Ranunculi Ternati Radix (Maozhaocao) clear liver fire, reduce swelling and dissipate nodules; Citri Reticulatae Pericarpium Viride (Qingpi) and Citri Reticulatae Pericarpium (Chenpi) soothe liver qi, resolve stagnation and harmonize the middle burner; Gardeniae Fructus (Zhizi) drains excess liver fire; Alismatis Rhizoma (Zexie) clears heat, promotes diuresis and directs heat downward. The whole formula soothes the liver and purges fire, nourishes yin and dissipates masses, regulates qi and harmonizes the middle burner, thus restoring smooth qi movement, quelling hyperactive liver fire, replenishing yin and blood, and clearing phlegm-heat. This formula may be associated with reduced thyroid hormone synthesis and release, potentially through attenuation of thyroidal autoimmune stimulation; however, this mechanism was not directly tested in the included RCTs. It may modulate inflammatory pathways including NLRP3/NF-κB, potentially alleviating local inflammatory infiltration and autoimmune injury in the thyroid gland; however, this pathway-level mechanism was not directly assessed in the included RCTs (29, 30).

Modified Xiaoyao Powder adopts the core therapeutic principles of soothing liver and relieving stagnation, strengthening spleen and nourishing blood, resolving phlegm and dissipating masses, astringing yin and harmonizing nutrient phase, targeting the pathogenesis of liver stagnation with spleen deficiency, qi stagnation and phlegm coagulation, yin-blood insufficiency, and internal disturbance of deficient fire. The ingredients collectively soothe liver qi, strengthen spleen and resolve phlegm, nourish blood and astringe yin, soften hardness and dissipate nodules, so that liver qi is dispersed, spleen function is reinforced, phlegm coagulation is resolved, yin-blood is replenished, and deficient fire is subdued. (31) This formula may be associated with improvement in thyroid-function indices, including reductions in elevated FT3 and FT4 and recovery of TSH. Because TSH recovery may lag behind changes in circulating thyroid hormones during GD treatment, TSH-related findings should be interpreted cautiously (32, 33). It may contribute to improvement in autoimmune activity, as reflected by reductions in TRAb and TPOAb levels; however, the present clinical data do not allow confirmation that MXYP directly restores immune homeostasis or prevents thyroid follicular-cell injury. (28) Xiaoyao Powder focuses on soothing liver qi, strengthening spleen and harmonizing nutrient phase, regulating qi and blood, and restoring yin–yang balance, addressing the pathogenesis of liver qi stagnation, spleen dysfunction, disharmony of qi and blood, and yin–yang imbalance to restore systemic homeostasis. This formula may be associated with recovery of TSH regulation; however, short-term TSH changes should be interpreted with caution because HPT-axis recovery in GD may be delayed and may not fully reflect long-term thyroid-function stability (20, 23, 32).

Modified Huagan Decoction (MHD) showed the highest SUCRA ranking for TRAb reduction in the present network meta-analysis. This formula clears liver fire, nourishes yin and dissipates masses, and regulates immune harmony, resulting in quenched fire, replenished yin, resolved phlegm, dispersed nodules, and restored yin–yang equilibrium. Because TRAb-mediated TSHR activation is central to GD pathogenesis, a reduction in TRAb may be clinically relevant to decreased thyroid stimulation and improvement of thyroid hormone levels; however, whether MHD directly inhibits autoimmune responses requires further mechanistic validation. (34) Huotan Jiangni Formula (HJF) showed the highest SUCRA ranking for TPOAb reduction in the present network meta-analysis. This formula follows the principles of expelling phlegm and directing counterflow downward, regulating qi and dissipating nodules, strengthening spleen and resolving phlegm, and harmonizing qi movement, targeting the pathogenesis of phlegm turbidity obstruction, disturbed qi movement, and binding of phlegm and qi, thus eliminating phlegm turbidity, smoothing qi flow, and resolving goiter. (19, 28) It may be associated with modulation of inflammatory signaling and cytokine-mediated immune activation, which have been implicated in autoimmune thyroid diseases; however, whether HJF directly reduces thyroid lymphocytic infiltration or follicular-cell injury in GD remains uncertain and requires further experimental validation. (28, 29) Whether HJF or other TCM formulations can reduce antithyroid drug-related liver dysfunction or leukopenia remains uncertain and requires further validation in rigorously designed clinical trials with standardized adverse-event monitoring (35, 36).

## 5 Clinical value and outlook

In conclusion, the integration of TCM with MMI may represent more than a simple “superposition of drug effects” and may provide a therapeutic framework combining targeted control and holistic regulation. This integrative approach harnesses the complementary strengths of Chinese and Western medicine in addressing the pathophysiology of GD. In recent years, this combination has become a prominent focus of research, showing potential advantages in enhancing short-term efficacy, while its effects on adverse events and long-term prognosis require further confirmation. It also provides a promising direction for future management of GD. However, several challenges remain, including inconsistencies in TCM-based pattern differentiation of GD, limited mechanistic exploration, and insufficient high-quality clinical evidence. These limitations restrict the broader clinical integration of TCM therapies with MMI. Future advancements in medical research are expected to overcome these limitations, enabling this therapeutic strategy to benefit a wider patient population and serving as a valuable model for integrative approaches in autoimmune disease management.

## 6 Strengths and limitations of this study

This study possesses several notable strengths. A comprehensive search was conducted across eight English and Chinese databases, including studies published up to June 2025, thereby minimizing the risk of omission. In total, fifteen RCTs involving 1,317 patients with GD were included, providing a relatively broad evidence base for the analysis. Moreover, the study strictly adhered to the PRISMA guidelines and the Cochrane Handbook for Systematic Reviews, ensuring methodological rigor and minimizing the potential for bias. Furthermore, a network meta-analysis model was employed to facilitate both direct and indirect comparisons among different “TCM + MMI” combinations. The efficacy of the interventions was quantified using SUCRA values, offering an evidence-based reference for clinical decision-making and contributing to the optimization of therapeutic strategies for GD.

However, several limitations should also be acknowledged. First, all included RCTs are short-term efficacy trials (4 weeks to 6 months), which is considerably shorter than the standard 12–18 month thionamide course used in Western GD management to assess long-term remission and relapse. These trials were not designed to evaluate time to euthyroidism or recurrence after drug withdrawal, which are conventional endpoints in Western endocrine trials.

Second, all patients were newly diagnosed with overt hyperthyroidism at baseline; no stably euthyroid patients on maintenance thionamide therapy were included, which represents a distinct study population from most Western GD trials. The heterogeneity in the composition, dosage, and treatment duration of Chinese medicine formulations across studies may have affected the comparability and consistency of efficacy outcomes. In addition, most included trials were single-center and small-sample studies, and some results relied on indirect comparisons, which could reduce the accuracy of the conclusions. The relatively short follow-up periods also limited the assessment of long-term disease control and thyroid function maintenance. Moreover, most outcome indicators were biochemical and lacked evaluation of patient-centered endpoints such as quality of life and symptom improvement. Finally, visual inspection of the funnel plots did not show obvious asymmetry; however, publication bias could not be formally excluded because quantitative tests were not performed and unpublished negative results may exist. In addition, AE reporting was incomplete and heterogeneous across the included trials, with inconsistent definitions, monitoring procedures, and severity grading; therefore, safety outcomes could only be summarized descriptively.

Therefore, future research should aim to standardize intervention protocols, incorporate patient stratification designs, and extend follow-up durations to strengthen the clinical evidence base and enhance the applicability of findings in real-world settings.

## 7 Conclusion

This study employed network meta-analysis to systematically evaluate the clinical efficacy of 14 TCM plus MMI regimens. The findings suggest that TCM plus MMI may be more effective than MMI monotherapy in improving thyroid function and reducing selected autoantibody levels, particularly TRAb and TPOAb, while the safety profile remains uncertain because of incomplete and inconsistent AE reporting. Despite the potential clinical relevance of these findings, limitations remain due to the heterogeneity of study designs, small sample sizes, and inadequate long-term follow-up. To further validate the efficacy and safety of TCM combined with MMI, higher-quality, multicenter, long-term randomized controlled trials are necessary. Such studies should aim to clarify the optimal treatment duration and potential mechanisms of action, as well as to facilitate the standardization and internationalization of this intervention strategy for the treatment of GD.

## Data Availability

The original contributions presented in the study are included in the article/**Supplementary Material**. Further inquiries can be directed to the corresponding authors.

## References

[1] Prasek K Płazińska MT Królicki L . Diagnosis and treatment of Graves’ disease with particular emphasis on appropriate techniques in nuclear medicine. General state of knowledge. Nucl Med Rev Cent East Eur. (2015) 18:110–6. doi: 10.5603/NMR.2015.0026 26315874

[2] Taylor PN Albrecht D Scholz A Gutierrez-Buey G Lazarus JH Dayan CM . Global epidemiology of hyperthyroidism and hypothyroidism. Nat Rev Endocrinol. (2018) 14:301–16. doi: 10.1038/nrendo.2018.18 29569622

[3] Streetman DD Khanderia U . Diagnosis and treatment of Graves disease. Ann Pharmacother. (2003) 37:1100–9. doi: 10.1345/aph.1C299 12841824

[4] Yang Z Zhao N Li J Wu Z Ma J . Effect of traditional Chinese medicine on Graves’ disease: a network meta-analysis. Front Pharmacol. (2024) 15:1411459. doi: 10.3389/fphar.2024.1411459 39239642 PMC11374712

[5] Rouse B Chaimani A Li T . Network meta-analysis: an introduction for clinicians. Intern Emerg Med. (2017) 12:103–11. doi: 10.1007/s11739-016-1583-7 27913917 PMC5247317

[6] Higgins JPT Altman DG Gotzsche PC Juni P Moher D Oxman AD . The Cochrane Collaboration’s tool for assessing risk of bias in randomised trials. BMJ. (2011) 343:d5928–8. doi: 10.1136/bmj.d5928 22008217 PMC3196245

[7] Cumpston M Li T Page MJ Chandler J Welch VA Higgins JP . Updated guidance for trusted systematic reviews: a new edition of the Cochrane Handbook for Systematic Reviews of Interventions. Cochrane Database Systematic Rev. (2019) 10:ED000142. doi: 10.1002/14651858.ED000142 31643080 PMC10284251

[8] Jackson D Riley R White IR . Multivariate meta-analysis: potential and promise. Stat Med. (2011) 30:2481–98. doi: 10.1002/sim.4172 21268052 PMC3470931

[9] Salanti G Ades AE Ioannidis JPA . Graphical methods and numerical summaries for presenting results from multiple-treatment meta-analysis: an overview and tutorial. J Clin Epidemiol. (2011) 64:163–71. doi: 10.1016/j.jclinepi.2010.03.016 20688472

[10] Chaimani A Higgins JPT Mavridis D Spyridonos P Salanti G . Graphical tools for network meta-analysis in STATA. PLoS One. (2013) 8:e76654. doi: 10.1371/journal.pone.0076654 24098547 PMC3789683

[11] Jiang P Chen W Feng L . Network meta-analysis of various surgical approaches for the treatment of posterolateral tibial plateau fractures. Injury. (2025) 56:112457. doi: 10.1016/j.injury.2025.112457 40449183

[12] McGuinness LA Higgins JPT . Risk-of-bias visualization (robvis): an R package and Shiny web app for visualizing risk-of-bias assessments. Res Synth Methods. (2021) 12:55–61. doi: 10.1002/jrsm.1411 32336025

[13] Yang H Bi X Tang H Zeng J Cong Y Wu T . Clinical efficacy of Yingliu treatment for Graves disease. Int J Clin Exp Med. (2015) 8:6145–53. PMC4483829 26131218

[14] Yang H Cong Y Wu T Tang H Ma M Zeng J . Clinical efficacy of Yingliu mixture combined with metimazole for treating diffuse goitre with hyperthyroidism and its impact on related cytokines. Pharm Biol. (2017) 55:258–63. doi: 10.1080/13880209.2016.1260595 27927064 PMC6130729

[15] Zhao J . Clinical observation on the therapeutic efficacy of integrated traditional Chinese and Western medicine in the treatment of Graves’ hyperthyroidism. In: Chinese Scientific and Technological Journal Database (Abstract Edition): Medicine and Health Care (2016). p. 00209. doi: 10.23736/s0031-0808.24.05155-3

[16] Shi L . Clinical study on Ziyin Xiaoying formula in the treatment of hyperthyroidism (Graves’ disease) with syndrome of liver–kidney yin deficiency and toxin damaging collaterals. In: Changchun University of Chinese Medicine. Changchun: Changchun University of Chinese Medicine (2017). Available online at: https://kns.cnki.net/KCMS/detail/detail.aspx?dbcode=CMFD&dbname=CMFD201701&filename=1016283525.nh (Accessed July 12, 2025).

[17] Zhao Y . Clinical study on Xiehuo Xiaoying formula combined with methimazole for treating hyperthyroidism at the initial treatment stage with syndrome of excessive heart and liver fire (2017). Available online at: https://www.cqvip.com/doc/degree/1869493934 (Accessed July 12, 2026).

[18] Wang X . Clinical observation on the effect of Shugan Xiaojiu formula on thyroid function in patients with hyperthyroidism (2018). Available online at: https://www.cqvip.com/doc/degree/1869372727 (Accessed July 12, 2026).

[19] Ji Z . Clinical observation on the therapeutic efficacy of Huotan Jiangni formula combined with methimazole in the treatment of Graves’ disease. Chin Natl Folk Med. (2018) 27:98–100+104.

[20] Jia L . Clinical observation on the therapeutic efficacy of Longmu Xiaoyao powder in the treatment of Graves’ disease of the excessive heart–liver fire type. In: Henan University of Chinese Medicine. Zhengzhou: Henan University of Chinese Medicine (2019). Available online at: https://kns.cnki.net/KCMS/detail/detail.aspx?dbcode=CMFD&dbname=CMFD201901&filename=1018309065.nh (Accessed July 12, 2025).

[21] Dai Z Shen Z Zhou Z Chen D Zhang L Yao D . Retrospective study on the effect of Astragalus-based preparations on serum thyrotropin receptor antibody (TRAb) levels in patients with Graves’ disease. Shanghai J Traditional Chin Med. (2020) 54(1):52–55

[22] Tian Z . Clinical observation on the therapeutic efficacy of Sanjie Xiaoying decoction in the treatment of toxic diffuse goiter (Graves’ disease). J Yunnan Traditional Chin Med Materia Med. (2020) 41:50–3.

[23] Duan S Wang Y Peng S . Clinical efficacy of modified Xiaoyao powder combined with methimazole tablets in patients with Graves’ disease of Qi stagnation and phlegm coagulation syndrome. In: Journal of Hunan University of Chinese Medicine, vol. 43. (2023). p. 1283–9.

[24] Xia S Lan X Wu Q Hu J . Clinical observation on modified Huagan decoction in the treatment of Graves’ disease with hyperactive liver fire syndrome. Shanxi J Traditional Chin Med. (2023) 39:27–9. doi: 10.20002/j.issn.1000-7156.2023.06.010

[25] Liu T . Clinical study on the treatment of Graves’ disease (hyperactivity of heart and liver fire syndrome) based on the theory of dislocation of ministerial fire. In: Chengdu University of Traditional Chinese Medicine (2023).

[26] Xia S . Clinical observation on the therapeutic efficacy of modified Yueju pill in the treatment of Graves’ disease with phlegm–Qi stagnation syndrome. In: Hunan University of Chinese Medicine (2024).

[27] Huang W . Exploring the therapeutic efficacy and material basis of Haizao Yuhu decoction for the treatment of Graves’ disease based on network pharmacology and metabolomics (2024). Available online at: https://www.cqvip.com/doc/degree/3477270697 (Accessed July 12, 2026).

[28] He Q Dong H Gong M Guo Y Xia Q Gong J . New therapeutic horizon of Graves’ hyperthyroidism: treatment regimens based on immunology and ingredients from traditional Chinese medicine. Front Pharmacol. (2022) 13:862831. doi: 10.3389/fphar.2022.862831 35462920 PMC9020194

[29] Guo Q Wu Y Hou Y Liu Y Liu T Zhang H . Cytokine secretion and pyroptosis of thyroid follicular cells mediated by enhanced NLRP3, NLRP1, NLRC4, and AIM2 inflammasomes are associated with autoimmune thyroiditis. Front Immunol. (2018) 9:1197. doi: 10.3389/fimmu.2018.01197 29915579 PMC5994487

[30] Chen F Kawashima A Luo Y Kiriya M Suzuki K . Innate immune-modulatory activity of Prunella vulgaris in thyrocytes functions as a potential mechanism for treating Hashimoto’s thyroiditis. Front Endocrinol (Lausanne). (2020) 11:579648. doi: 10.3389/fendo.2020.579648 33304319 PMC7701117

[31] Nan Nan Wenzhi Hao Lin Li Yueyun Liu Jiaxu Chen . Bidirectional regulation of depression via the hypothalamic–pituitary–adrenal axis and gut–brain axis from the perspective of simultaneous treatment of the liver and spleen. In: Journal of Beijing University of Traditional Chinese Medicine, Beijing: Journal of Beijing University of Traditional Chinese Medicine vol. 48. (2025). Available online at: https://kns.cnki.net/KCMS/detail/detail.aspx?dbcode=CJFQ&dbname=CJFDLAST2025&filename=JZYB202507002 (Accessed March 14, 2026).

[32] Uy HL Reasner CA Samuels MH . Pattern of recovery of the hypothalamic-pituitary-thyroid axis following radioactive iodine therapy in patients with Graves’ disease. Am J Med. (1995) 99:173–9. doi: 10.1016/s0002-9343(99)80137-5 7625422

[33] Kahaly GJ Bartalena L Hegedüs L Leenhardt L Poppe K Pearce SH . 2018 European Thyroid Association guideline for the management of Graves’ hyperthyroidism. Eur Thyroid J. (2018) 7:167–86. doi: 10.1159/000490384 30283735 PMC6140607

[34] Smith TJ Hegedüs L . Graves’ disease. N Engl J Med. (2016) 375:1552–65. doi: 10.1056/NEJMra1510030 27797318

[35] Ioannidis JPA Evans SJW Gøtzsche PC O’Neill RT Altman DG Schulz K . Better reporting of harms in randomized trials: an extension of the CONSORT statement. Ann Intern Med. (2004) 141:781–8. doi: 10.7326/0003-4819-141-10-200411160-00009 15545678

[36] Vicente N Cardoso L Barros L Carrilho F . Antithyroid drug-induced agranulocytosis: state of the art on diagnosis and management. Drugs R D. (2017) 17:91–6. doi: 10.1007/s40268-017-0172-1 28105610 PMC5318340

