# Supplementary material for: Efficacy of methimazole combined with traditional Chinese medicine in the treatment of Graves’ disease: a systematic review and network meta-analysis
Source: Front Endocrinol (Lausanne). 2026 Jul 14;17:1767935. doi: 10.3389/fendo.2026.1767935 (PMC13407158; doi:10.3389/fendo.2026.1767935)
Supplement: Supplementary file 1 [file DataSheet1.pdf]

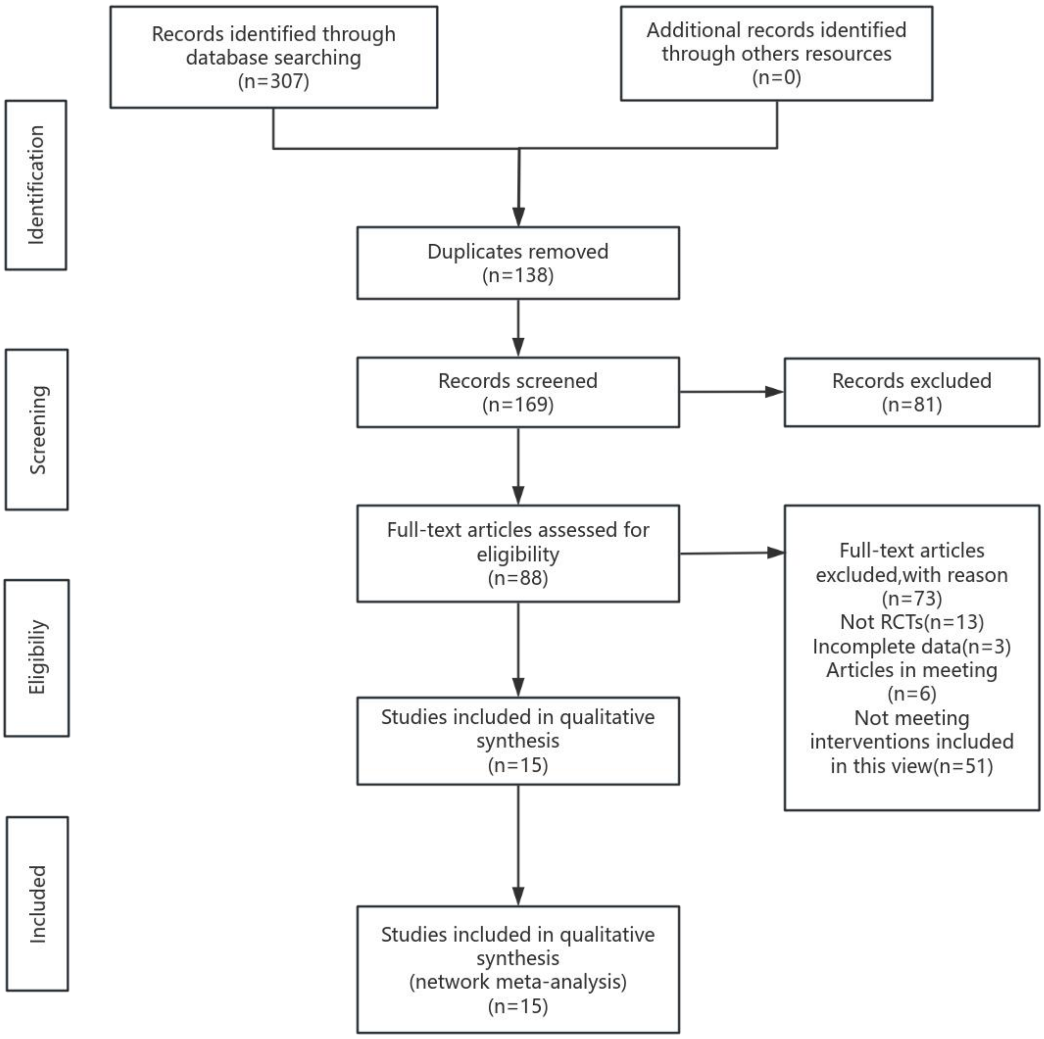

Supplementary Figure 1. Flow diagram of literature selection.

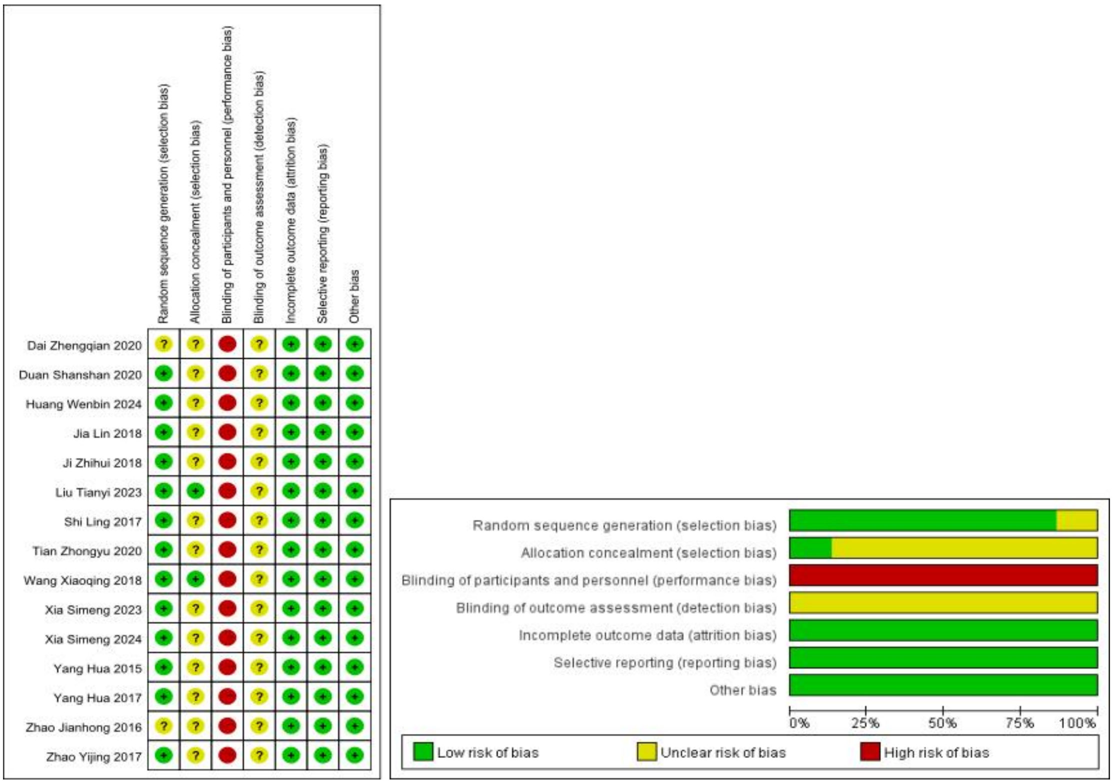

Supplementary Figure 2. Risk of bias graph for each included study.

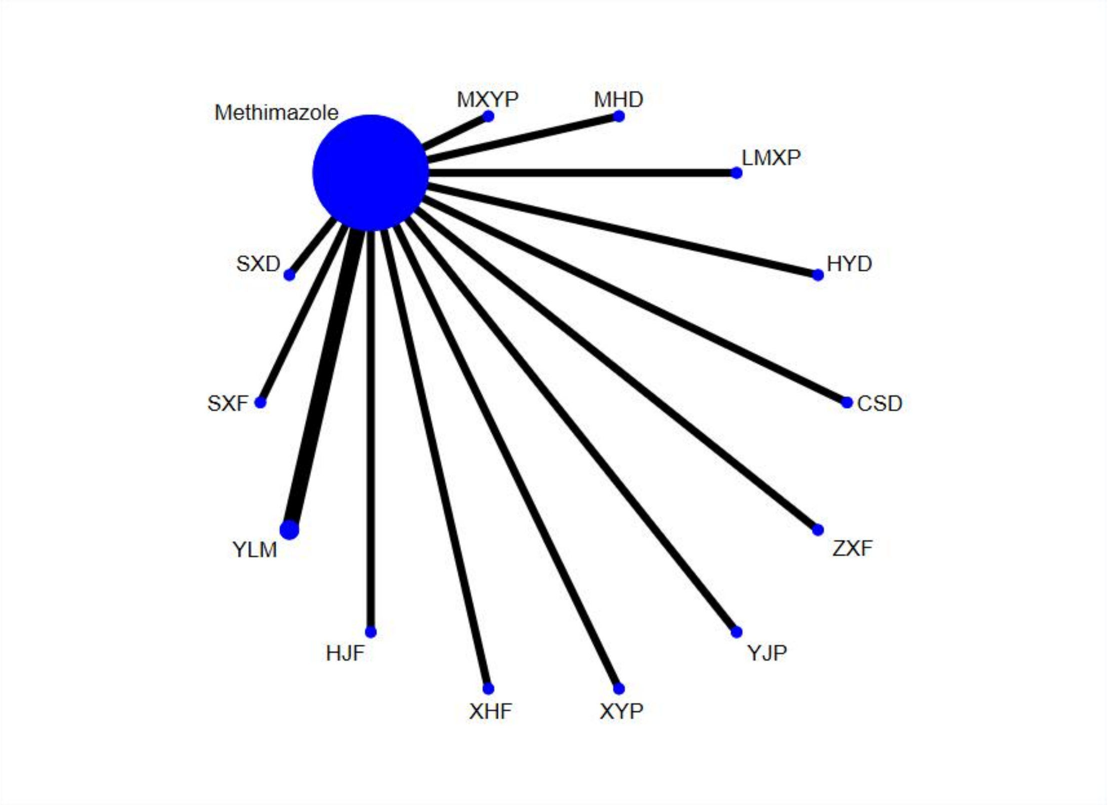

Supplementary Figure 3. A

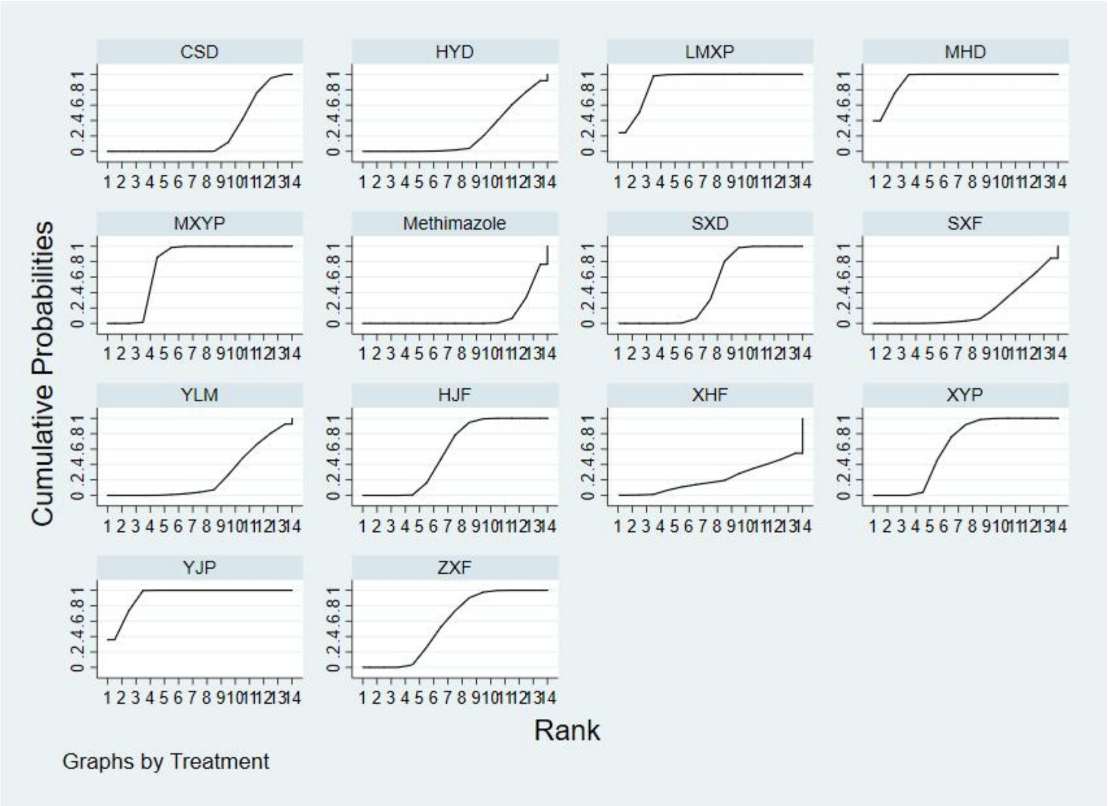

Supplementary Figure 3. B

Supplementary Figure 3. The evidence of natural extract to influence FT3. (A), network graph of the FT3. (B), the SUCRA plot for FT3.

Description: (A) Network diagrams are the most intuitive visualizations used to represent the relationships between various interventions. The size of the nodes and the thickness of the connecting lines were positively correlated with the number of studies. (B) The surface under the cumulative ranking curve (SUCRA) metric was used to rank the effectiveness of each treatment and identify the best treatment. The larger the area under the SUCRA curve, the better the treatment effect.

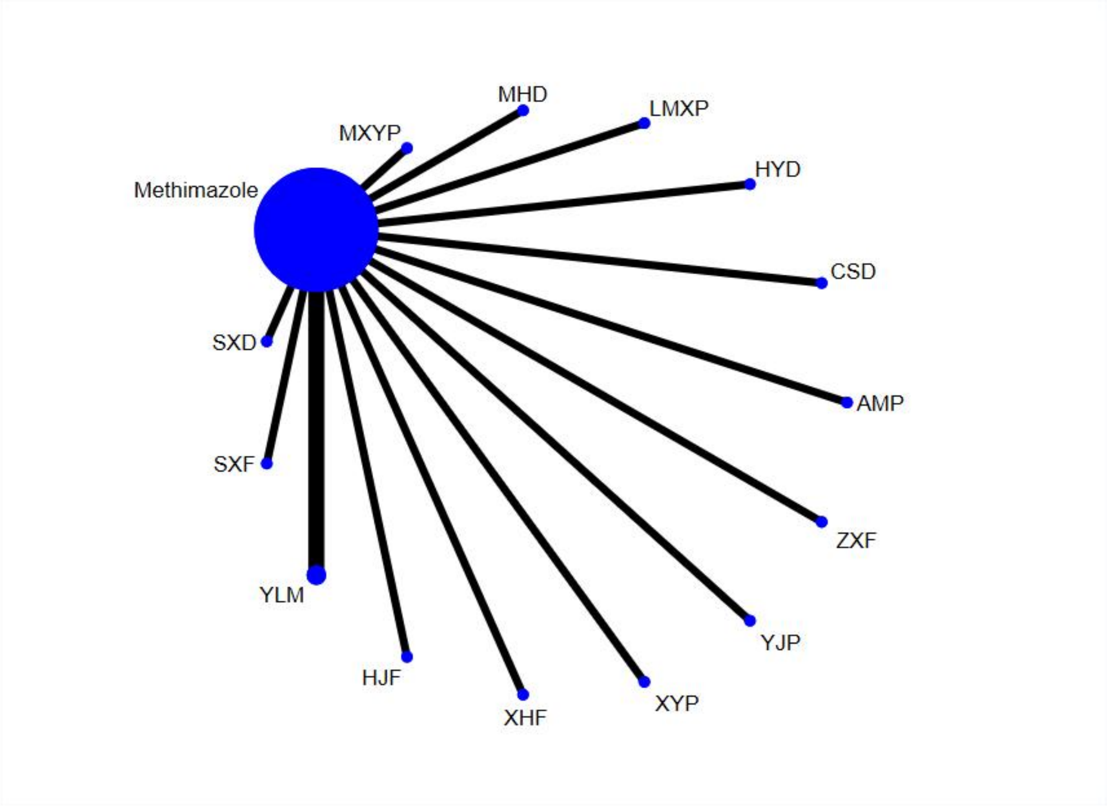

Supplementary Figure 4. A

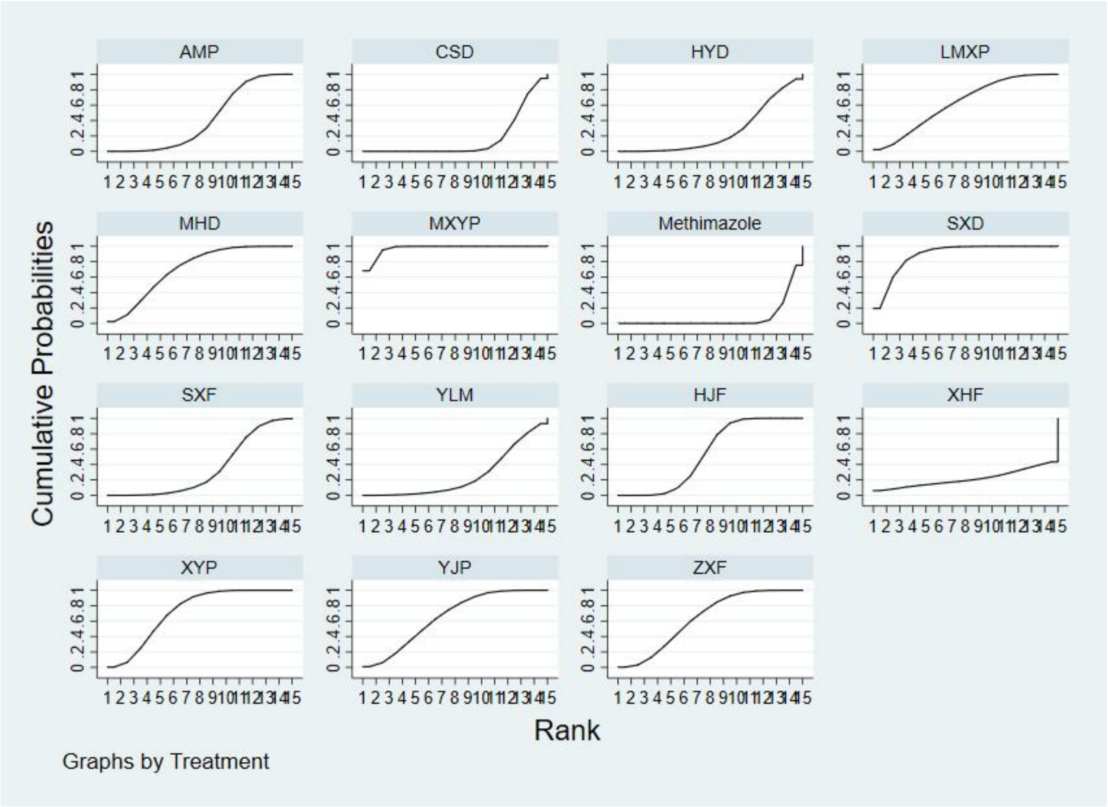

Supplementary Figure 4. B

Supplementary Figure 4. The evidence of natural extract to influence FT4. (A), network graph of the FT3. (B), the SUCRA plot for FT4.

Description: (A) Network diagrams are the most intuitive visualizations used to represent the relationships between various interventions. The size of the nodes and the thickness of the connecting lines were positively correlated with the number of studies. (B) The surface under the cumulative ranking curve (SUCRA) metric was used to rank the effectiveness of each treatment and identify the best treatment. The larger the area under the SUCRA curve, the better the treatment effect.

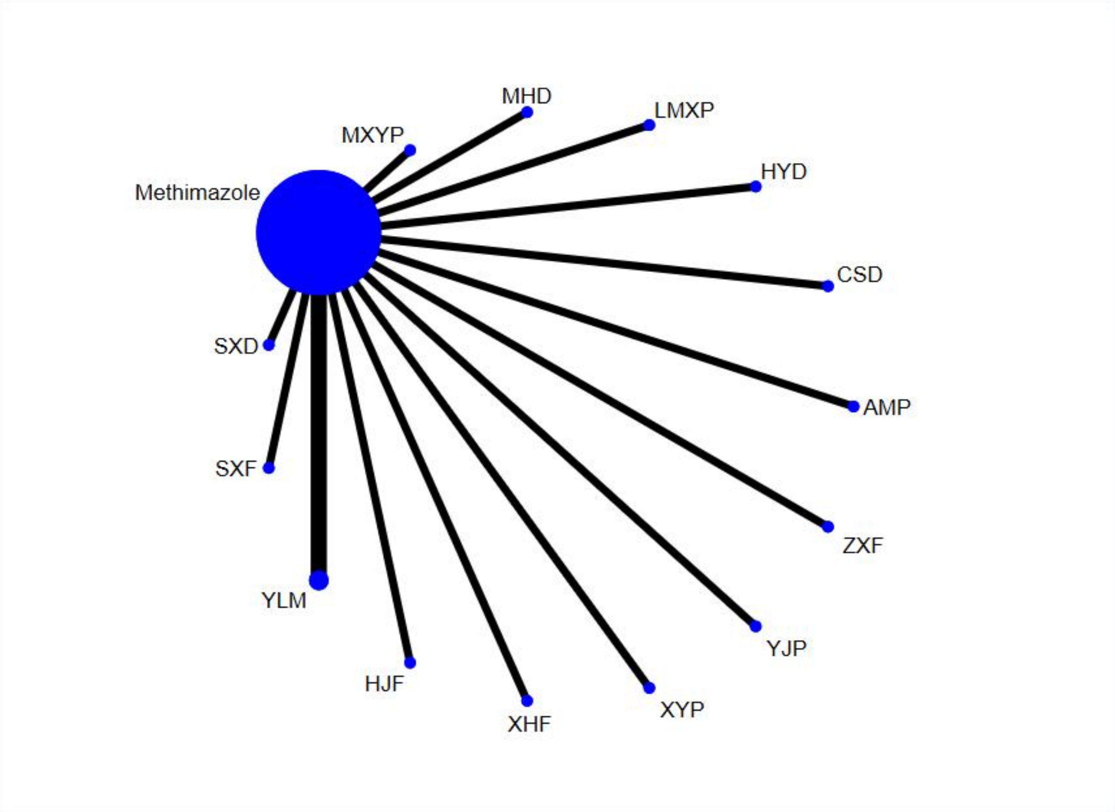

Supplementary Figure 5. A

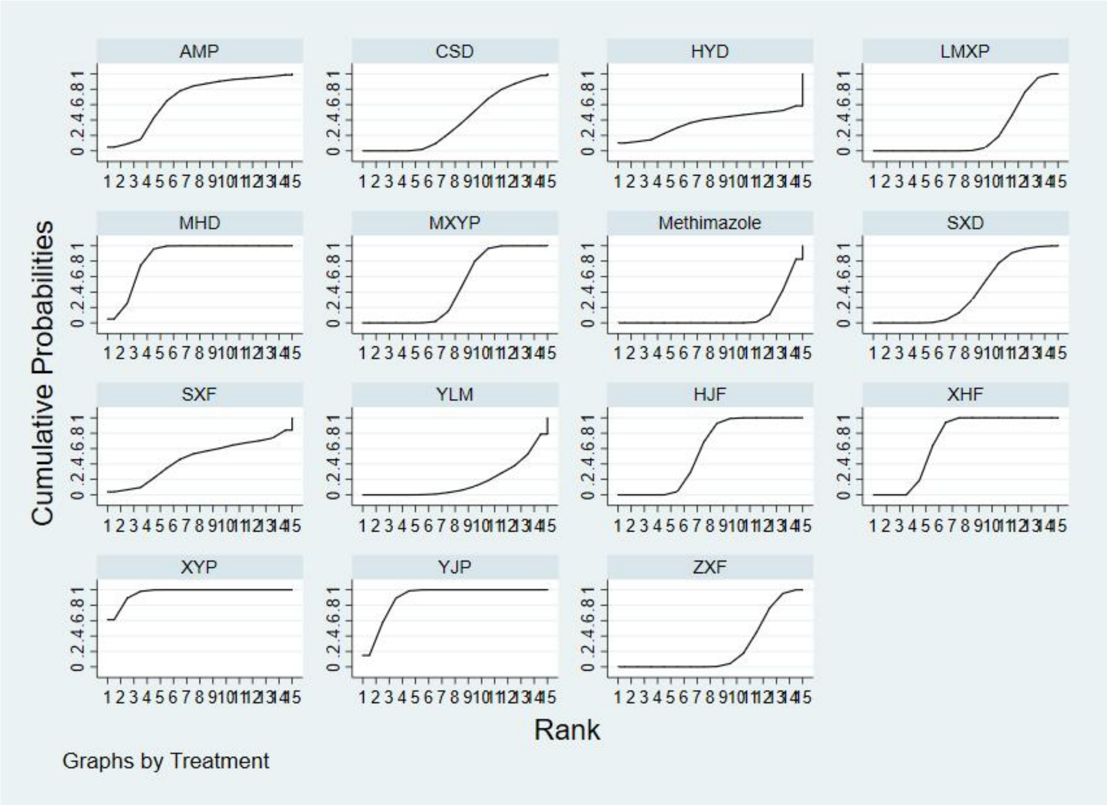

Supplementary Figure 5. B

The evidence of natural extract to influence TSH. (A), network graph of the TSH. (B),

the SUCRA plot for TSH.

Description: (A) Network diagrams are the most intuitive visualizations used to represent the relationships between various interventions. The size of the nodes and the thickness of the connecting lines were positively correlated with the number of studies. (B) The surface under the cumulative ranking curve (SUCRA) metric was used to rank the effectiveness of each treatment and identify the best treatment. The larger the area under the SUCRA curve, the better the treatment effect.

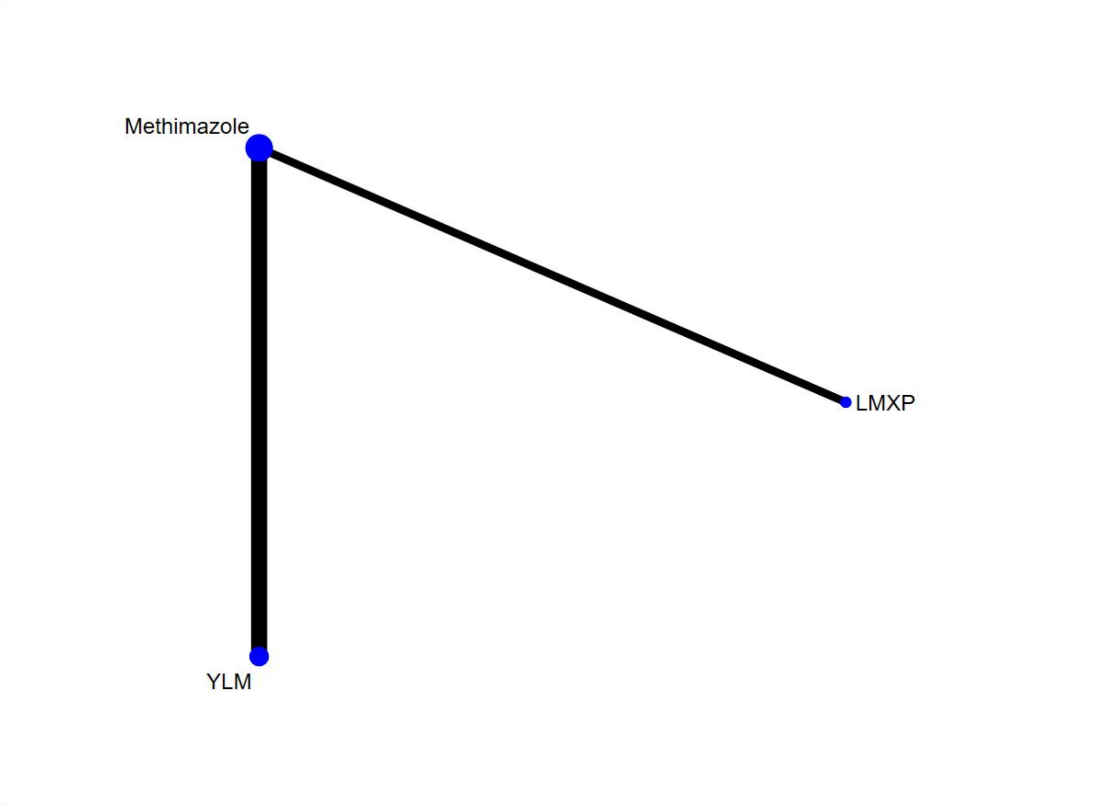

Supplementary Figure 6. A

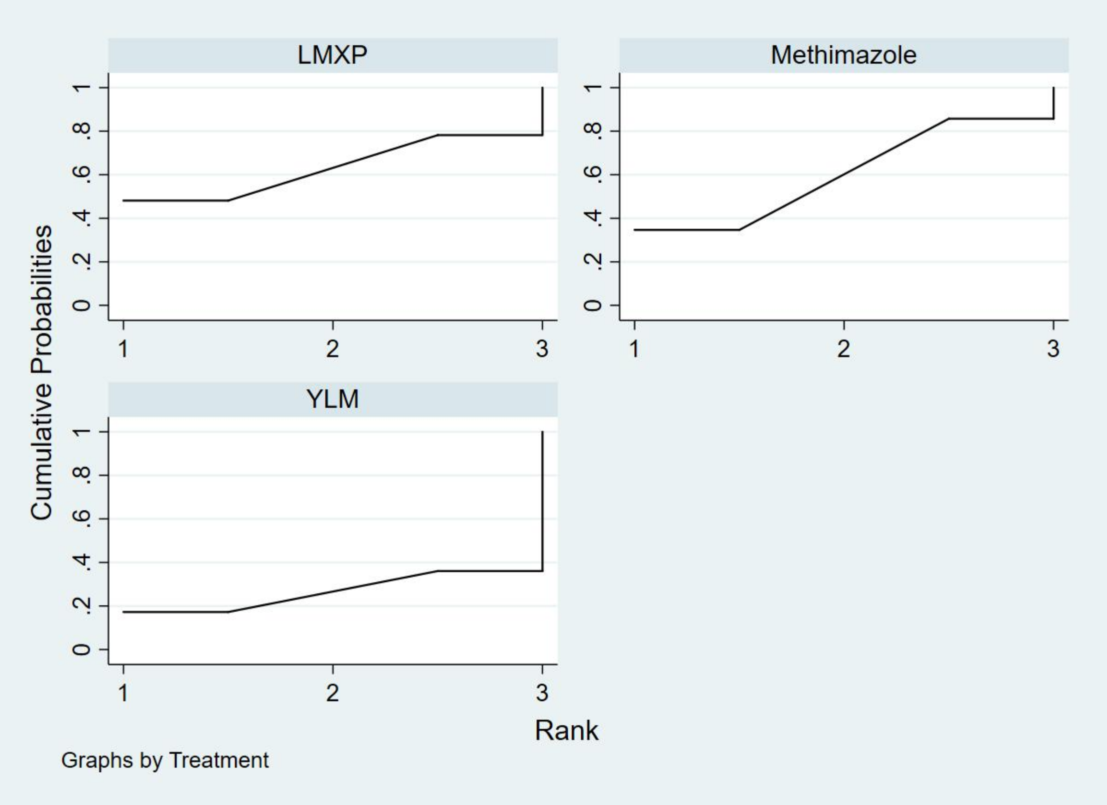

Supplementary Figure 6. B

Supplementary Figure 6. The evidence of natural extract to influence TgAb. (A), network graph of the TgAb. (B), the SUCRA plot for TgAb.

Description: (A) Network diagrams are the most intuitive visualizations used to represent the relationships between various interventions. The size of the nodes and the thickness of the connecting lines were positively correlated with the number of studies. (B) The surface under the cumulative ranking curve (SUCRA) metric was used to rank the effectiveness of each treatment and identify the best treatment. The larger the area under the SUCRA curve, the better the treatment effect.

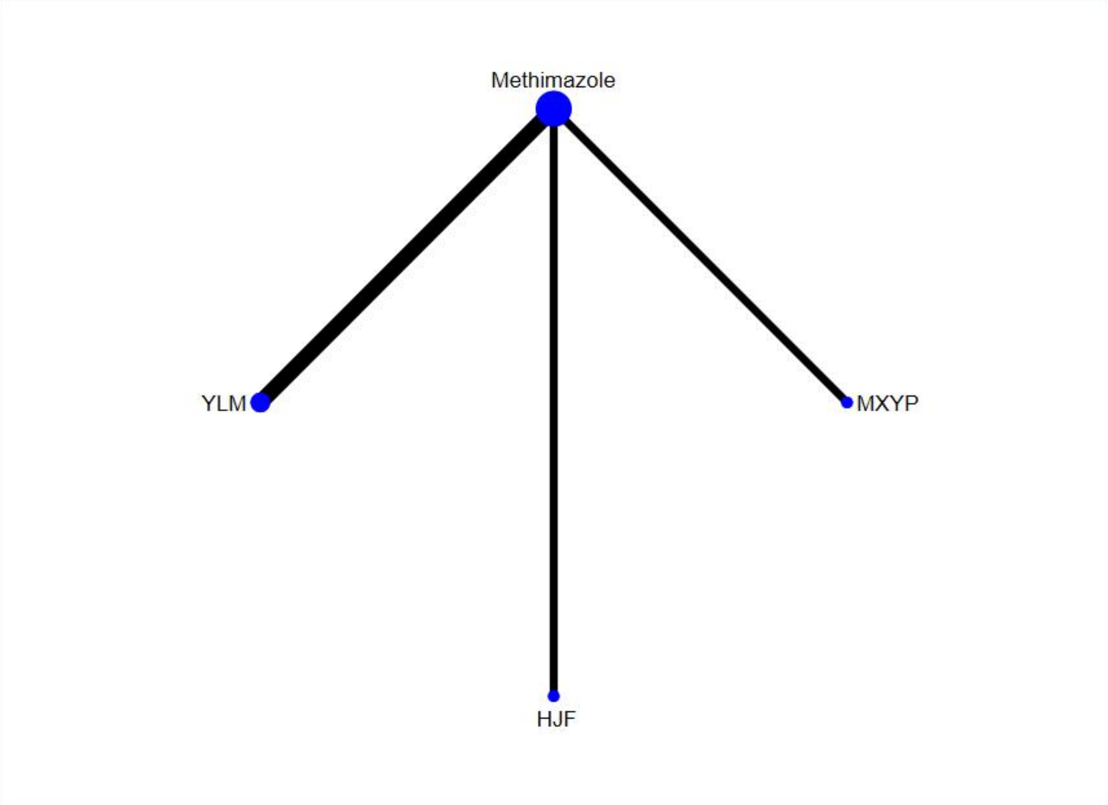

Supplementary Figure 7. A

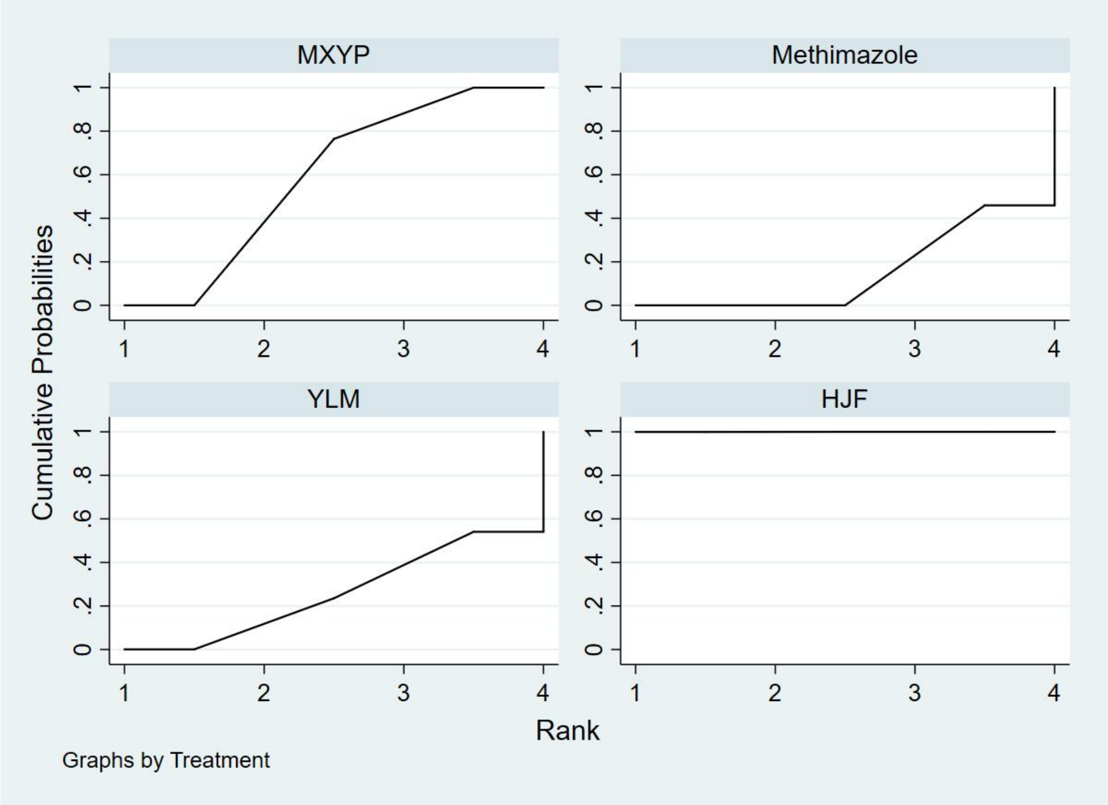

Supplementary Figure 7. B

Supplementary Figure 7. The evidence of natural extract to influence TPOAb. (A), network graph of the TPOAb. (B), the SUCRA plot for TPOAb.

Description: (A) Network diagrams are the most intuitive visualizations used to represent the relationships between various interventions. The size of the nodes and the thickness of the connecting lines were positively correlated with the number of studies. (B) The surface under the cumulative ranking curve (SUCRA) metric was used to rank the effectiveness of each treatment and identify the best treatment. The larger the area under the SUCRA curve, the better the treatment effect.

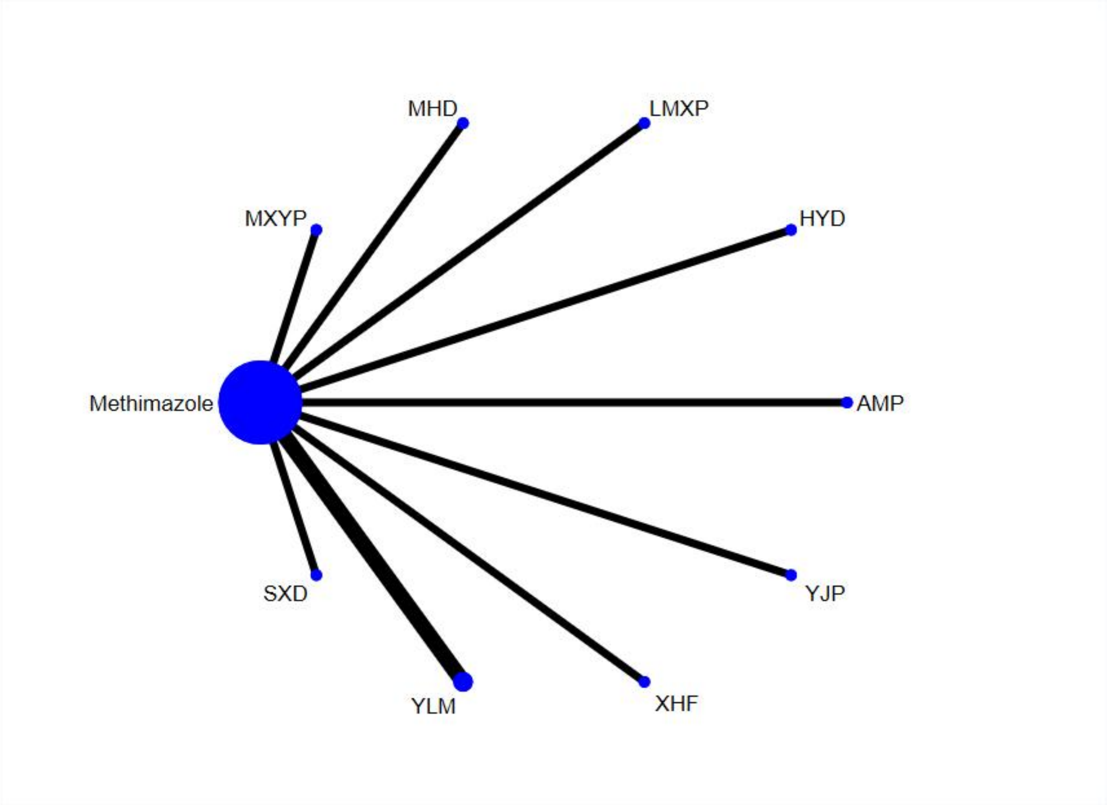

Supplementary Figure 8. A

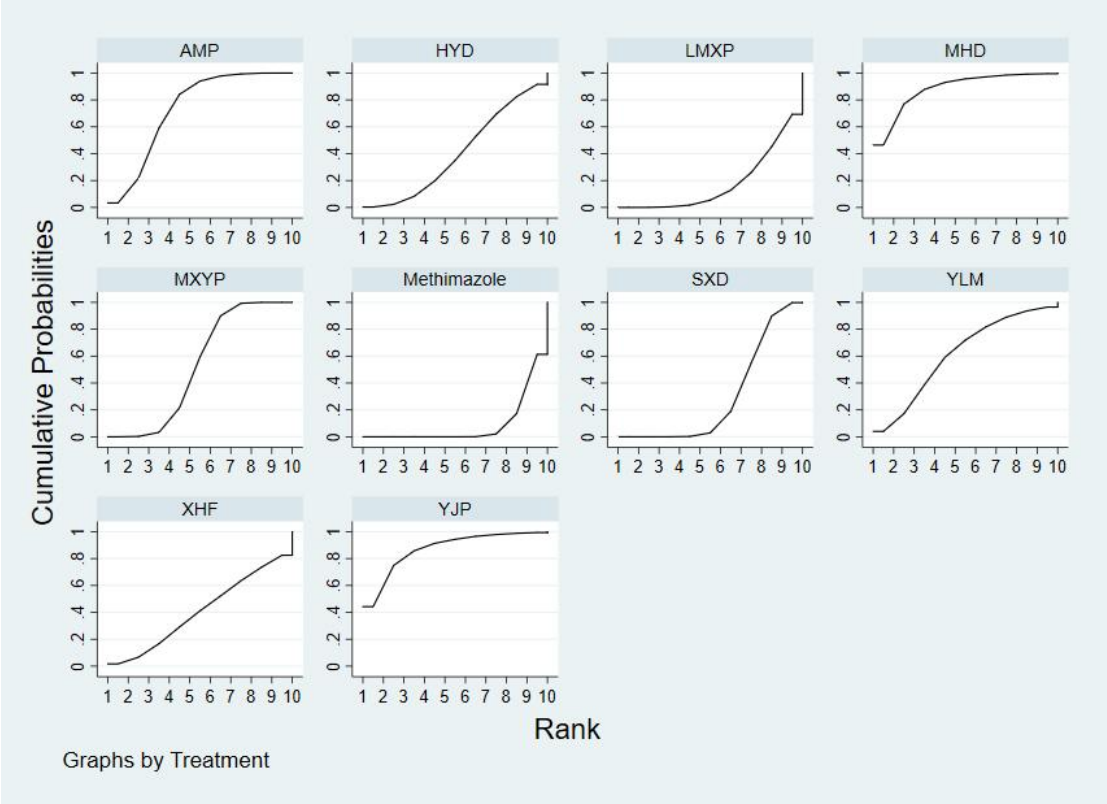

Supplementary Figure 8. B

Supplementary Figure 8. The evidence of natural extract to influence TRAb. (A), network graph of the TRAb. (B), the SUCRA plot for TRAb.

Description: (A) Network diagrams are the most intuitive visualizations used to represent the relationships between various interventions. The size of the nodes and the thickness of the connecting lines were positively correlated with the number of studies. (B) The surface under the cumulative ranking curve (SUCRA) metric was used to rank the effectiveness of each treatment and identify the best treatment. The larger the area under the SUCRA curve, the better the treatment effect.

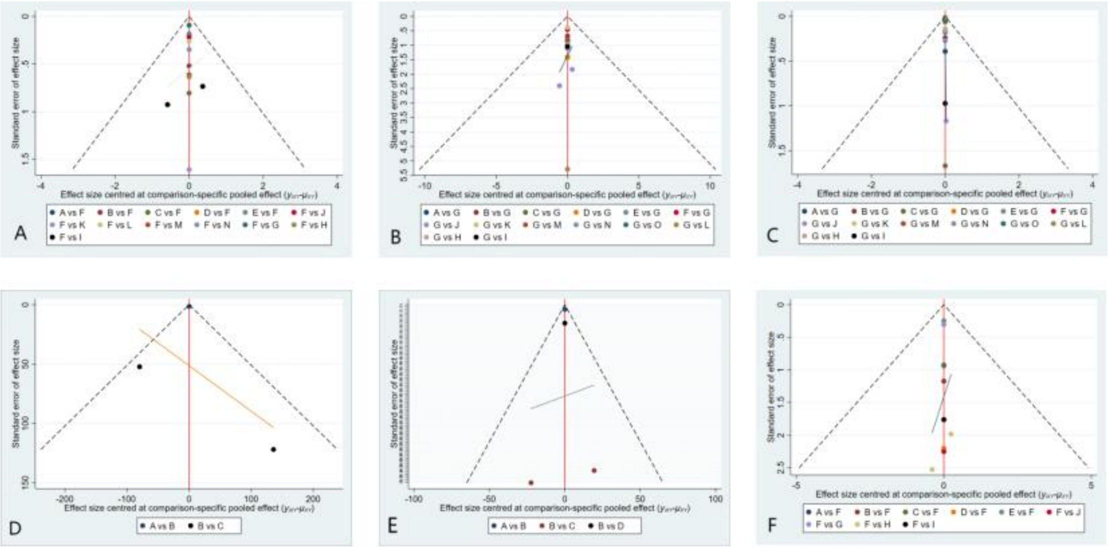

Supplementary Figure 9. Funnel plot on publication bias. (A): FT3; (B): FT4; (C): TSH; (D): TgAb; (E): TPOAb; (F): TRAb
